# Multiomics analysis dissects the molecular foundation of perianal fistulas associated with Crohn’s disease and of cryptoglandular origin

**DOI:** 10.1093/ecco-jcc/jjag080

**Published:** 2026-06-30

**Authors:** Céline Mamie, Daniela Cabalzar-Wondberg, Matthias Turina, Marcin Wawrzyniak, Benjamin Misselwitz, Nicola Zamboni, Claudia Gottier, Silvia Lang, Gerhard Rogler, Alvaro Avivar-Valderas, Olga de la Rosa, Ninfa Candela, Jay Tang, Yasser Morsy, Michael Scharl

**Affiliations:** Department of Gastroenterology and Hepatology, University Hospital Zurich, University of Zurich, Zurich 8091, Switzerland; Department of Visceral and Transplant Surgery, University Hospital Zurich, University of Zurich, Zurich 8091, Switzerland; Department of Visceral and Transplant Surgery, University Hospital Zurich, University of Zurich, Zurich 8091, Switzerland; Department of Gastroenterology and Hepatology, University Hospital Zurich, University of Zurich, Zurich 8091, Switzerland; Department of Medicine II, University Hospital LMU Munich, Munich 80539, Germany; Institute of Molecular Systems Biology, ETH Zurich, Zurich 8093, Switzerland; PHRT Swiss Multi-Omics Center, ETH Zurich, Zurich 8093, Switzerland; Department of Gastroenterology and Hepatology, University Hospital Zurich, University of Zurich, Zurich 8091, Switzerland; Department of Gastroenterology and Hepatology, University Hospital Zurich, University of Zurich, Zurich 8091, Switzerland; Department of Gastroenterology and Hepatology, University Hospital Zurich, University of Zurich, Zurich 8091, Switzerland; Cell Therapy Technology Sciences, Takeda Cell Therapy Technology Center (Tigenix SAU), Madrid 28760, Spain; Cell Therapy Technology Sciences, Takeda Cell Therapy Technology Center (Tigenix SAU), Madrid 28760, Spain; Takeda Development Center Americas (TDCA), Takeda Development Center Americas, Inc, Cambridge, MA 02142, United States; Takeda Development Center Americas (TDCA), Takeda Development Center Americas, Inc, Cambridge, MA 02142, United States; Department of Gastroenterology and Hepatology, University Hospital Zurich, University of Zurich, Zurich 8091, Switzerland; Department of Gastroenterology and Hepatology, University Hospital Zurich, University of Zurich, Zurich 8091, Switzerland

**Keywords:** basic science, experimental models and pathophysiology, microbiology

## Abstract

**Background and objective:**

Perianal fistulas, either of cryptoglandular origin (CgF) or associated with Crohn’s disease (CDF), have limited treatment options and pose a tremendous burden for affected patients. We recently showed that the epithelial–mesenchymal transition (EMT) contributes to CDF pathogenesis, but detailed mechanisms need further evaluation. Here, we performed multiomics analysis to gain further molecular insights into fistula pathogenesis.

**Design:**

Rectal biopsies, swabs, fistula curettage, and serum samples were derived from patients with either CDF (*n* = 23) or CgF (*n* = 17) and analyzed by bulk RNA sequencing, metagenomics, untargeted metabolomics, or multiplex-ELISA, where appropriate.

**Results:**

Transcriptomics revealed striking differences in gene expression between rectal mucosa and fistula tract samples. However, the transcriptomes of CDF and CgF were comparable, and genes involved in EMT, inflammation and tumor necrosis factor signaling were prominent in both fistula types. A set of 18 genes was found to be differentially expressed in CDF and CgF and might allow discrimination. The overall microbiome composition within fistula tracts did not differ between CDF and CgF patients, but there was a significant difference in rectal microbiome compositions. On a species level, we detected an enrichment of disease-specific, pathogenic species in the fistula tracts. Of note, *Bacteroides* ssp., *Fusobacterium animalis*, and *Staphylococcus aureus* prevailed within CDF.

**Conclusion:**

Our data demonstrate only minor differences in the transcriptome and the microbiome between CDF and CgF, but clear differences when compared to rectal mucosa biopsies. Thus, our data suggest that the molecular makeup underlying the pathophysiology of fistulas might be comparable between CDF and CgF.

## Graphical abstract

**Graphical Abstract:**
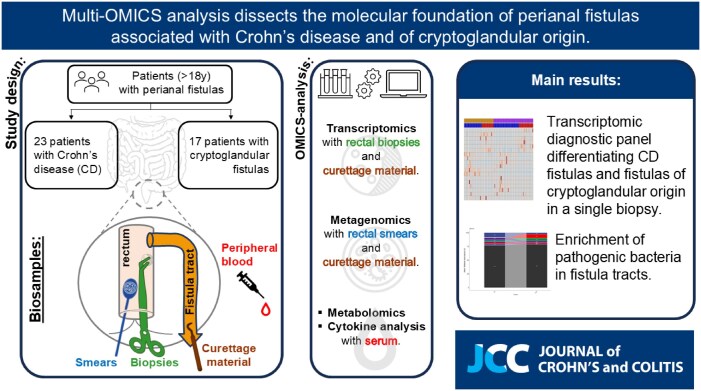

## Introduction

Perianal fistulas are mainly of cryptoglandular origin (CgF) or associated with Crohn’s disease (CDF), and are characterized by anorectal pain, malodorous pus discharge, and sometimes fecal incontinence.^1^ Those symptoms frequently represent a tremendous physical and psychological burden for afflicted patients, critically impacting their quality of life.^2–4^ Treatment options are still limited, and healing rates are low, particularly in patients with complex fistulas.^5^^,^^6^ Medical treatment options are frequently ineffective^7–9^ and surgical interventions are challenging due to the high risk of irreversible incontinence.^10^ Furthermore, both medical and surgical therapies are associated with frequent recurrences and do not provide a definitive solution in many patients.^11^^,^^12^

A recent literature review estimates the prevalence of perianal fistulas in Europe at 1.69 per 10 000 population, including CgF and CDF at 0.86 and 0.76 per 10 000 population, respectively.^13^ Anal fistulas can be the initial complication in 10% of patients with CD and at least 25% of patients will present perianal complications during disease course, and the prevalence of fistulizing disease is expected to increase drastically.^9^^,^^14^^,^^15^ Idiopathic fistulas mainly of cryptoglandular origin are the most common type of clinically diagnosed perianal fistulas, since 90% are believed to emerge from an acute anal gland infection leading to an abscess and eventually to fistula formation.^16^^,^^17^ Although this cryptoglandular theory was proposed and revised almost a century ago,^16^^,^^18^ the molecular mechanism leading to fistula formation has still not been fully elucidated.^19^^,^^20^ A description of fistula-in-ano subtypes according to the localization of the abnormal channel(s) in the perineum, put forward by Parks et al., laid the foundation for fistula classification.^21^ Moreover, the chronic luminal inflammation in CD exacerbates the condition, which can evolve to carcinoma.^22^

On a pathogenetic level, anal gland inflammation, intestinal inflammation, epithelial-to-mesenchymal transition (EMT), as well as a disturbed intestinal microbiota composition have been associated with fistula formation.^1^ We have previously presented evidence for a number of molecular processes resulting in the development of CDF:^23^ due to an epithelial barrier defect, bacteria can invade the intestinal mucosa and activate an inflammatory response. Increased expression of (1) inflammatory molecules, such as tumor necrosis factor (TNF), TGF-beta or IL-13, (2) matrix-remodeling molecules, such as matrix metalloproteinase 9 (MMP9), and (3) cell invasiveness-associated molecules such as beta-6-integrin induce the transformation of the intestinal epithelial cells (IECs) into myofibroblast-like cells that penetrate into deeper tissue layers.^24–26^ Those “transitional cells” lining the fistula tract still express epithelial markers (CK8, CK20), but are shaped as mesenchymal cells and do not undergo epithelial cell apoptosis. EMT is also characterized by transcription factors such as SNAIL, SLUG, or ETS-1, which are found to be increased along fistula tracts.^24^^,^^27^^,^^28^ In CgF, increased levels of IL-1-beta, IL-8, IL-12p40, and TNF have been described.^29^ In addition, increased levels of TGF-beta, vimentin, Zeb-1, and SNAIL as well as decreased levels of E-cadherin have been shown in CgF.^30^ Further, alterations in the microbiome composition within fistula tracts have been shown.^31^^,^^32^ An abnormal inflammatory response to microbiome components that invade the intestinal mucosa (either due to an infected anal gland or to the defective mucosal barrier) might trigger fistula formation.^33^ In addition, the microbiome might contribute to maintaining inflammation and to preventing fistula healing.

Here, we aimed to decipher the transcriptome and the microbiome within the tracts of either CgF or CDF and to identify potential molecular (dis)similarities. Unraveling the molecular details of fistulas might finally allow us to pinpoint new targets for medical intervention to improve fistula healing.

## Methods

### Ethics

The studies were conducted in accordance with the Declaration of Helsinki. Ethical approval was obtained from the Cantonal Ethics Committee of the Canton Zürich, Switzerland (KEK-ZH 2016-01310, KEK-ZH-Nr. 2016-00367, KEK-ZH-Nr. 2020–02402, KEK-ZH-Nr. 2020-01768, PB_2019-00169, PB_2022-02295).

### Patient enrollment

Patients undergoing fistula surgery at the University Hospital Zurich, Switzerland, were informed and when consenting signed a written informed consent. The therapy procedures are not relevant for this study but can be found in the respective references. Fistulodesis, a minimal invasive treatment to close perianal fistulas, was performed on 23 fistulizing patients either with CD in remission or without inflammatory bowel disease (IBD), between 2017 and 2018 within the framework of an investigator-initiated trial.^34^ A further 17 patients with CD were undergoing Darvadstrocel stem cell therapy between 2019 and 2022 within clinical routine treatment.^35^^,^^36^ Altogether, 23 patients with CDF and 17 patients with CgF aged between 19 and 82 years for whom biomaterial was available were included in the study. In the CDF group, 60% were males with an average age of 38 years, whereas in the CgF group, 53% were males with an average age of 46 years. An overview of both patient cohorts is shown in Table 1. Patient demographics and fistula description according to Parks classification^21^ as determined by the surgeons are listed in Table S1. Where available, the branching category^37^ is noted.

**Table 1.** Summary of demographics and fistula description

	Patients with CDF	Patients with CgF
**Total patients included**	23	17
**Gender: male, % (*n*)**	60% (12)	53% (9)
**Age at intervention, mean, years**	38	46
**Age at first fistula manifestation, mean, years**	32	NA
**Years with CD, mean**	12	NA
**Years with fistula, mean**	7	NA
**Modified Parks classification:**		
** Transphinteric**	21	16
** Suprasphinteric**	2	0
** Intersphincteric**	2	0
** Extrasphincteric**	1	0
** Superficial**	3	0
** Unknown**	0	3
**Fistula type:**		
** Single-unbranched**	12	12
** Branched**	4	0
** Multiple (2 single-unbranched)**	4	2
** Unknown**	0	3
**Total number of fistula tracts**	34	19
**Number of fistula tracts measured**	23	14
**Fistula tract length, mean, cm**	4.1	3.5
**Percentage of fistulas > 4 cm (*n*)**	47.8 (11)	42.8 (6)

Patients with fistulizing disease included in this study presented either Crohn’s disease (CDF) associated fistula(s) or fistula(s) of cryptoglandular origin (CgF). Both patient groups are compared by gender, age at intervention and at first fistula manifestation, the number of years with CD, and with fistula by calculating the mean. The fistulas are described using the modified Parks classification (trans-/supra-/extra-/intrasphinteric, or superficial). In addition, the type of fistulas, either single-unbranched or single-branched or multiple, located in one patient, is described. Average fistula length is 4 cm in both patient groups. NA: non available.

### Patient samples

Biosamples (peripheral blood mononuclear cells [PBMCs], serum, smears from rectum, feces, rectal biopsies, and curettage material from the fistula tracts) and clinical data from 40 patients were collected as stated in the ethically approved study protocol (Table S1). The biomaterial was processed and stored as quickly as possible. Biopsies and curettage material were either snap frozen (native) or stored in RNALater™ (cat. AM7020, Life technologies, USA). Fecal samples, collected in OMNIgene GUT (cat. OM-200, DNAgenotek, Canada) and ESwab™ (cat. 480/481CE, Copan group, Italy) were stored at −80°C.

### Multiomics analyses

Details on the methods for RNA sequencing (RNA-seq), differential gene expression (DEG), metagenomics, metabolomics, and cytokine analysis by multiplex-ELISA are given in the supplement material. We also conducted a structured sensitivity assessment of available demographic covariates (age and gender) across our RNA-seq and metagenomic analyses (Fig. S1).

## Results

### Transcriptomic analysis reveals different gene expression profiles in rectal biopsies and fistula tracts

**Figure 1.**
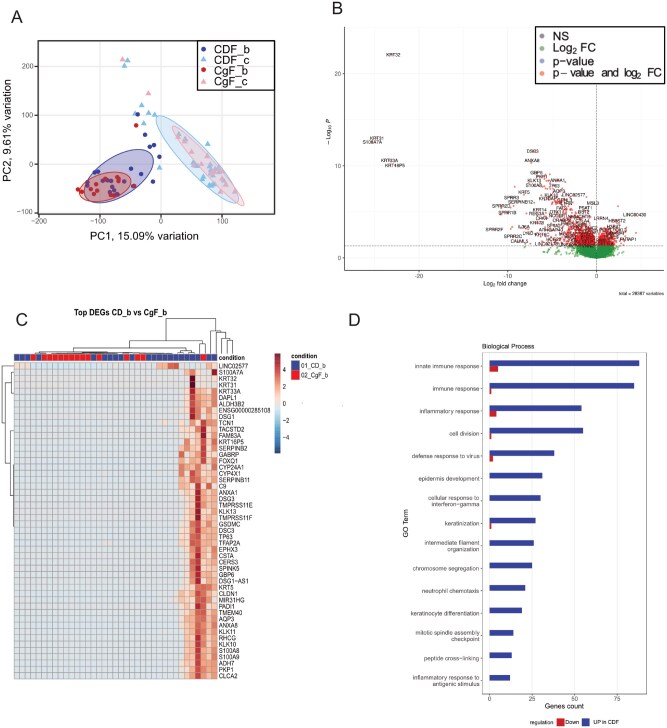
The transcriptomic profiles of fistulas derived from Crohn’s disease (CDF) or of cryptoglandular origin (CgF) are comparable and differ from the rectal mucosal transcriptome. (A) Principal component analysis (PCA) showing the geometric distance between rectal biopsies (circle) and curettage samples (triangle) from patients with CDF(*n* = 23, blue) and CgF (*n* = 17, red). For the rectal biopsies, each dot represents a patient sample (except one with two biopsies); for the curettage material each dot represents a fistula curettage sample. (B) Volcano plot of the differentially expressed genes (DEGs) comparing CDF (*n* = 22) biopsies to CgF (*n* = 15) biopsies (significant DEGs = 1974 out of 28 387 genes detected, adjusted *P*-value ≤ .05). (C) Heatmap of the 50 top DEGs. (D) Gene ontology terms of the significant biologic processes using 1974 DEGs. Genes involved in innate immune, inflammatory responses, and cell division are upregulated in patients with CD.

We analyzed the gene expression profiles of rectal mucosa biopsy samples (CDF = 22, CgF = 15) and fistula curettage samples (CDF = 32 and CgF = 18) by bulk RNA-seq. While only one rectal biopsy was analyzed per patient (except for one patient with two), several fistula tract curettage samples (two or three) were analyzed from patients with branched fistulas or with multiple single fistulas. Principal component analysis (PCA) revealed a separate clustering of the gene expression profiles of rectal biopsy and fistula curettage samples, indicating different global gene expression profiles (Figure 1A). However, we did not detect such a difference with respect to the underlying diseases of the respective fistulas.

When comparing the gene expression profile between CDF and CgF within the biopsies derived from the rectal mucosa, we detected 1974 significantly DEGs (*P-*value adjusted < .05) out of 28 387 features (Figure 1B). Rectal mucosa from patients with CDF compared to patients with CgF exhibited high RNA expression levels of pro-inflammatory mediators of S100 family members such as S100A7A, S100A8, and S100A9 (Figure 1C) as well as of genes involved in inflammation resolution such as ANXA1 and ANXA8. A further striking finding was the increased expression of genes involved in epithelial barrier homeostasis and integrity, such as keratins (KRT32, KRT31, KRT33A, KRT16P5), small-prolin-rich proteins (SPRR3, SPRR2D, SPRR1B, SPRR2F, SPRR2C), SERPINS (SERPINB2, SERPINB11), TP63, cell–cell adhesion molecules (CLDN1 claudin, DSG1, DSG3) and PKP1 in CDF mucosa compared to CgF mucosa (Figure 1B, C). Gene Ontology (GO) analysis shows a significant enrichment of functional GO terms mainly related to immune and inflammatory responses and cell division in CDF biopsies when compared to CgF biopsies (Figure 1D). These data probably highlight the difference between the chronic inflammatory state and the high cellular turnover in the mucosa of CD patients compared to non-CD patients. Similarly, GO analysis on cellular components (Figure S2A) and molecular functions (Figure S2B) also highlighted the upregulated cellular turnover in the rectal biopsies of CDF compared to CgF.

**Figure 2.**
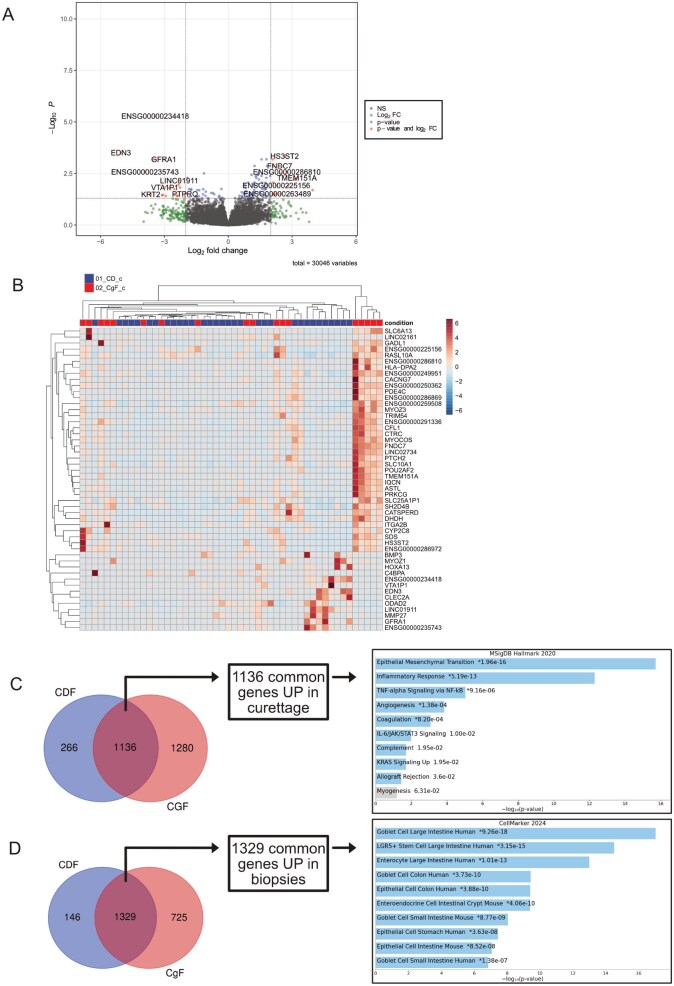
Perianal fistulas of cryptoglandular (CgF) and Crohn’s disease (CDF) origin are transcriptionally similar, expressing markers for epithelial–mesenchymal transition (EMT), inflammatory response, and TNF signaling. (A) Volcano plot of the DEGs comparing curettage samples from CDF (*n* = 32) to CgF (*n* = 18) (significant DEGs = 129 out of 30 046 genes, adjusted *P*-value ≤ .05). (B) Heatmap of top 50 significant DEGs in curettage samples from CDF compared to CgF. No gene ontology terms could be determined, revealing no specific significantly different molecular pathways between the fistula gene expression profiles from CDF and from CgF. (C) Venn diagram combining 1402 upregulated DEGs (comparing biopsies to curettage in CDF) and 2416 upregulated DEGs (comparing biopsies to curettage in CgF). In total, 1135 genes are upregulated in curettage material and found in CDF and CgF fistula tracts (log_2_FC ≥ |2|; *P*-value adjusted < .001). Bar chart of top enriched terms from the Molecular Signatures Database (MSigDB) hallmark gene set analysis. The top 10 enriched terms for the input gene set are displayed based on the −log_10_(*P*-value), with the actual *P*-value shown next to each term. The term at the top has the most significant overlap with the input query gene set. (D) Venn diagram combining 1475 downregulated DEGs (comparing biopsies to curettage in CDF) and 2054 downregulated DEGs (comparing biopsies to curettage in CgF). In total, 1329 are downregulated in curettage and common to CDF and CgF fistula tracts. Due to the comparison performed, those genes also represent the 1329 upregulated DEGs in biopsies and common to CDF and CgF (log_2_FC ≥ |2|; *P*-value adjusted < .001). Bar chart of top enriched terms from the Cell_marker 2024 library. The top 10 enriched terms for the input gene set are displayed based on the −log_10_(*P*-value), with the actual *P*-value shown next to each term. The term at the top has the most significant overlap with the input query gene set.

RNA expression profiles derived from curettage material of patients with either CDF or CgF detected 129 DEGs (*P-*value adjusted < .05) out of 30 046 variables (Figure 2A). The unsupervised clustering of DEGs found within the fistula tracts of patients with CgF and CDF did not indicate a disease-specific pattern (Figure 2B). Also, GO term analysis on this set of DEGs did not uncover any significant differences. Similarly, the PCA of all curettage samples did not reveal any clear clusters depending on underlying disease, Parks classification, or branching categories (Figure S3). Nevertheless, curettage samples derived from the same patient clustered together.

### Perianal CDF and CgF express markers of EMT, inflammatory responses, and TNF signaling

We subsequently determined the expression level of genes that are intrinsic to fistula tissues in both diseases. First, we compared DEGs between biopsies and curettage material in patients with CDF (Figure S4A). We found 1475 downregulated and 1402 upregulated DEGs in curettage material compared to biopsies from patients with CDF (log_2_FC ≥ |2|; *P-*value adjusted < .001). Second, we identified 2054 downregulated and 2416 upregulated DEGs in curettage material compared to biopsies in patients with CgF (log_2_FC ≥ |2|; *P-*value adjusted < .001). In a Venn diagram we showed 1136 genes that were upregulated in curettage material (compared to biopsies) in both patients with CDF and CgF (Figure 2C). Similarly, we identified 1329 commonly upregulated genes in biopsy samples (compared to curettage) in both patients with CDF and CgF (Figure 2D). Of note, the genes found to be upregulated in curettage are the same genes that are found to be downregulated in biopsies, and vice versa, due to the comparison performed here (Figure S4B).

The resulting gene lists were then analyzed on the Enrichr platform (supplementary material). Based on the set of 1136 upregulated genes in curettage, the Molecular Signatures Database (MSigDB) hallmark gene set analysis highlighted the EMT, inflammatory response, and TNF signaling via NF-kB pathways as most prominent, and these processes have previously been associated with fistula development (Figure 2C). On the other hand, the 1329 upregulated genes in the biopsies reflected the activated cellular processes: compared to the Cellmarker 2024 library, the genes were representative of processes found in goblet cells, Lgr5+ stem cells, and enterocytes, and, to no great surprise, those cells were identified as the most abundant (Figure 2D). For consistency, the results from MSigDB and Cellmarker 2024 analysis for both gene lists are shown in Figure S4C.

Since EMT has been described as a prominent pathogenetic feature in the pathogenesis of perianal fistulas, we further examined the 46 EMT-related genes from the MSigDB list (Figure S5A–D) in addition to 45 EMT-related genes proposed by Yang et al. in the guidelines for research in EMT^38^ (Figure S6A–D). The core EMT transcription factors (TFs) SNAI1, SNAI2, TWIST1, ZEB1, and ZEB2 were upregulated in fistula curettage samples compared to biopsies in both fistula types. Furthermore, additional TFs associated with EMT, such as ERG, ETS1, GSC (Goosecoid), KLF6, NFKB1, PRRX1, SOX4, and TCF4, were also found to be upregulated in fistula curettage samples compared to biopsies. Epithelial markers that are typically lost during EMT, such as CDH1 (E-cadherin), EPCAM, or OCNL (occludin), were downregulated, whereas mesenchymal markers such as FN1 (fibronectin) and VIM (vimentin) were upregulated in curettage samples compared to biopsies. Finally, TGFB1, a main driver of EMT, and HIF1-alpha were upregulated in curettage samples compared to rectal biopsies. These data highlight the importance of EMT-related genes in the formation and maintenance of perianal CDF and CgF.

### A limited number of 18 genes are differentially expressed between patients with CDF and CgF

**Figure 3.**
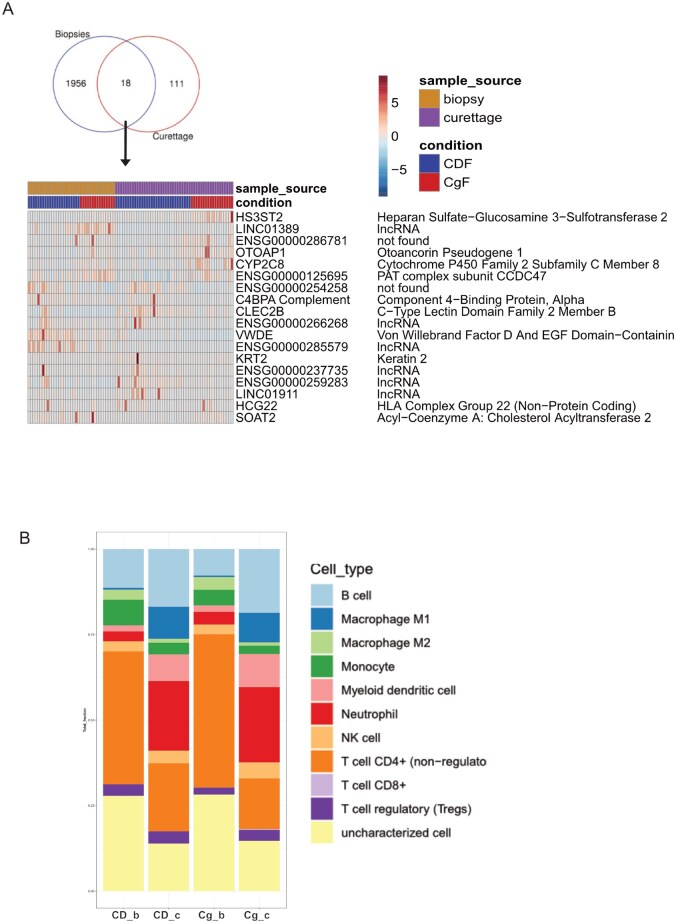
Transcriptomic analysis reveals 18 differentially expressed genes (DEGs) detectable in cryptoglandular (CgF) and Crohn’s disease (CDF) patients in biopsy and curettage material. (A) Venn diagram of DEGs in biopsy and curettage samples from patients with CDF compared to biopsy and curettage samples of CgF origin (*P*-value adjusted < .05). Intersection corresponds to down- and upregulated genes significantly differentially expressed (CDF vs CgF) in biopsies as well as in curettage. List of 18 common DEGs in biopsy and curettage material of CDF compared to CgF, including six long non-coding RNAs (lncRNAs). (B) Graphical representation of immune cell type proportions in biopsies and curettage samples of CDF and CgF after immune deconvolution analysis on bulk RNA-seq data. M1 macrophages and neutrophils are more represented in curettage whereas CD4+ T cells (non-regulatory) are more represented in biopsies independent of the origin of the fistulizing disease.

To uncover the difference between CDF and CgF, we combined the lists of DEGs (CDF vs CgF, *P-*value adjusted < .05) from the rectal biopsies (1974 genes, Figure 1B) and from the fistula curettage samples (129 genes, Figure 2A). A Venn diagram shows the 18 genes that were differently expressed in both the biopsies and the curettage material between patients with CDF and patients with CgF (Figure 3A). To ensure correctness, we performed the same analysis on the lists of genes that were upregulated and downregulated (Figure S7A, B): six genes were downregulated in both tissues from patients with CDF compared to those with CgF (HS3ST2, LINC01389, ENSG00000286781, OTOAP, CYP2C8, PAT complex subunit CCDC47) while 11 genes were upregulated in the same comparison (C4BPA, CLEC2B, VWDE, KRT2, HCG22, ENSG00000254258, five long non-coding RNAs) (Figure 3A). The only gene differently expressed in curettage versus biopsies was SOAT2, which was downregulated in curettage samples from patients with CDF compared to CgF and upregulated in biopsies from patients with CDF compared to CgF. The unique expression pattern of these 18 genes may allow discrimination of CgF and CDF with a simple rectal mucosal biopsy in patients with fistula as a first manifestation.^39^ We used published transcriptomics data from Rizzo et al. to test the validity of our gene expression panel on curettage and biopsy tissues.^40^ In testing the discrimination power using the published cohort analysis comprising nine patients with CDF and six patients with CgF, we found that the curettage material (area under the curve [AUC] = 0.704) and the rectal biopsies (AUC = 0.815) showed similar discrimination power (Figure S8). Therefore, we could validate our finding using external and published data that a single rectal biopsy might discriminate between a patient with a CD fistula and a patient with a cryptoglandular fistula.

We next profiled the immune cell populations present within the rectal mucosa and the fistula tracts by applying cell deconvolution on the RNA-seq data. Here, we again found differences depending on the anatomical localization of the tissues. In contrast, the observed differences between the underlying fistula etiologies were less pronounced (Figure 3B). CD4+ T (non-regulatory) cells were significantly more present in rectal biopsies than in fistulas, whereas the fistulas contained more M1 macrophages and neutrophils than rectal biopsies in both CDF and CgF (Figure S9). However, we did not find any significant differences in any immune cell population between CDF and CgF fistulas. These data emphasize the significant differences in the immune cell composition between rectal biopsies and fistula tracts regardless of the underlying disease.

### The microbiota in CDF tracts is similar to that in CgF, but is characterized by a dysbiotic rectal microbiome

**Figure 4.**
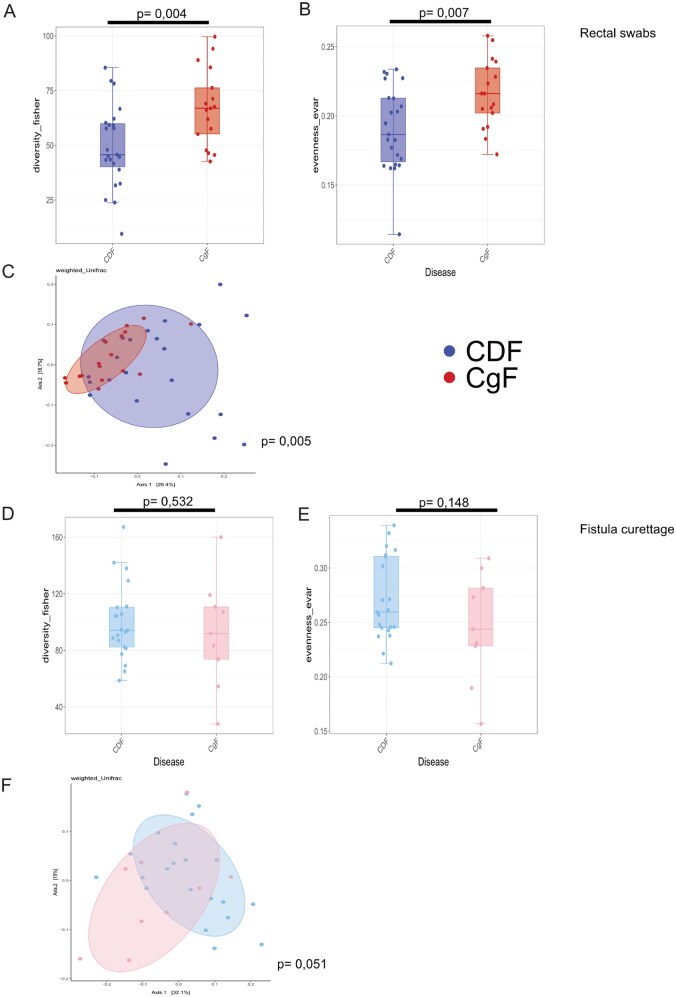
Metagenomics analysis reveals a different composition of the microbiome within the tracts of Crohn’s disease (CDF) associated and cryptoglandular (CgF) fistulas. Graphical representations of alpha-diversity by Fisher (A) and by Evar (B) in rectal swabs show a significant reduction of microbiome diversity and evenness in CDF compared to CgF. (C) Representation of beta-diversity by WeightedUniFrac in rectal swabs. Graphical representations of alpha-diversity by Fisher (D) and by Evar (E) in curettage material indicate no significant difference in diversity and evenness between CDF and CgF. Representation of beta-diversity by WeightedUniFrac (F) in curettage material (two-tailed Mann–Whitney U test, adjusted *P*-value). Data are shown as boxplots with median, interquartile range (IQR), and 1.5 × IQR whiskers; individual observations are overlaid with jitter.

To explore alterations of the microbiome composition in the rectum or within the fistula tracts of patients with CDF or CgF, we performed shotgun metagenomics on rectal swabs and fistula curettage material. When comparing the alpha-diversity in rectal swabs between CDF and CgF patients, we found significant differences, as demonstrated by Fisher’s diversity and Evar evenness analysis (Figure 4A, B). The composition of the rectal microbiome of patients with CD was characterized by a decreased diversity and lower richness compared to the rectal microbiomes of patients with CgF. A reduced microbial diversity is in fact a hallmark of the intestinal microbiome of patients with CD compared to non-IBD patients. Interestingly, the composition of the rectal microbial composition in individual patients with CD did not indicate homogeneous perturbations within this group, as shown by the dispersed samples in the beta-diversity plot (Figure 4C).

In contrast, there was no difference in diversity and richness of the microbiome in the fistula tracts of patients with CDF or CgF as assessed by Fisher’s diversity and Evar evenness analysis (Figure 4D, E). The individual fistula microbiomes of each patient, nevertheless, were rather heterogeneous within both cohorts (Figure 4F). PCA of all curettage samples did not reveal any clear clusters depending on the underlying disease, Parks classification, or branching categories. However, the microbiome profiles of multiple fistulas from the same patient clustered together (Figure S10).

### Accumulation of potentially pathogenic species in the fistula tracts of patients with CDF and CgF

**Figure 5.**
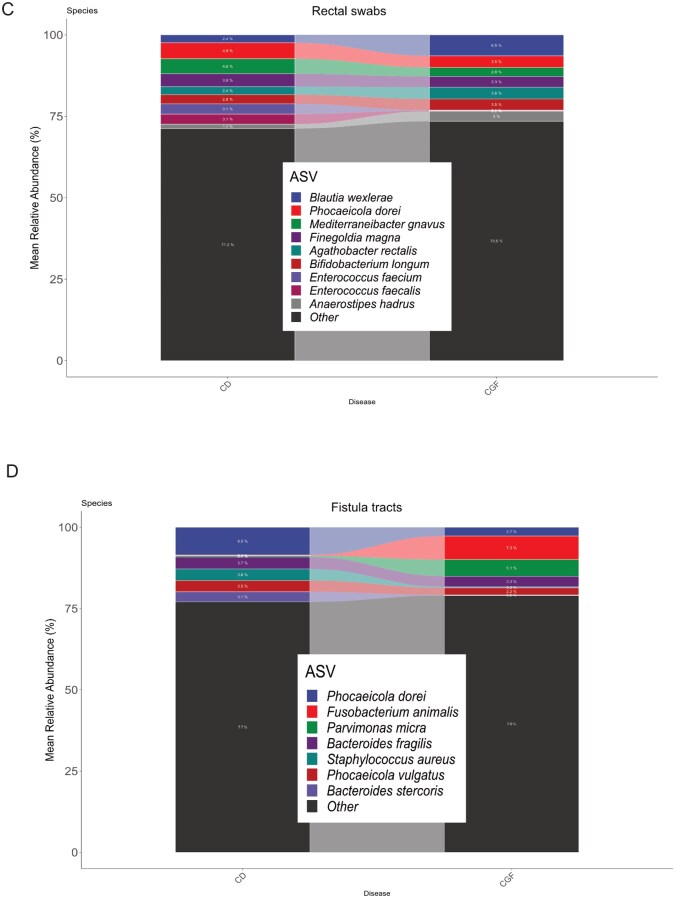

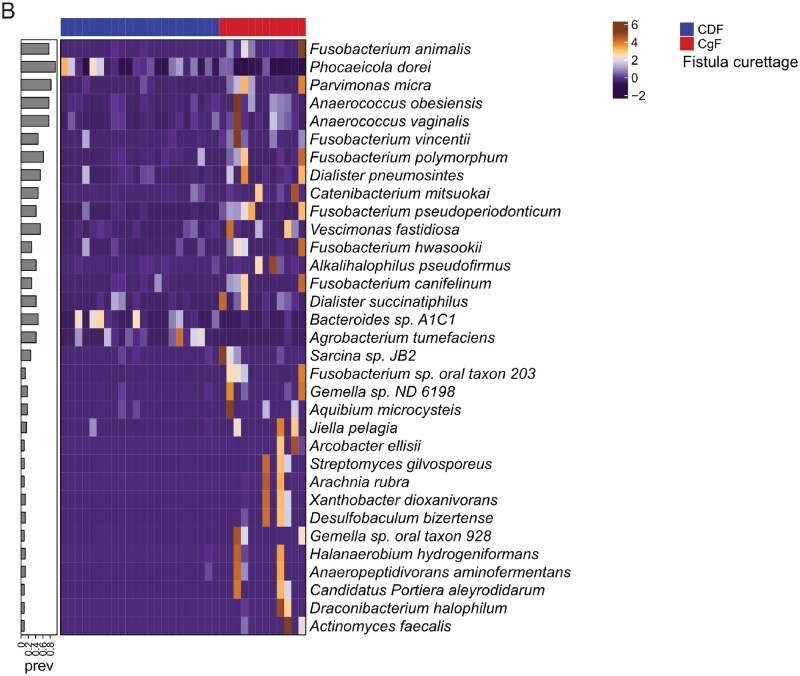

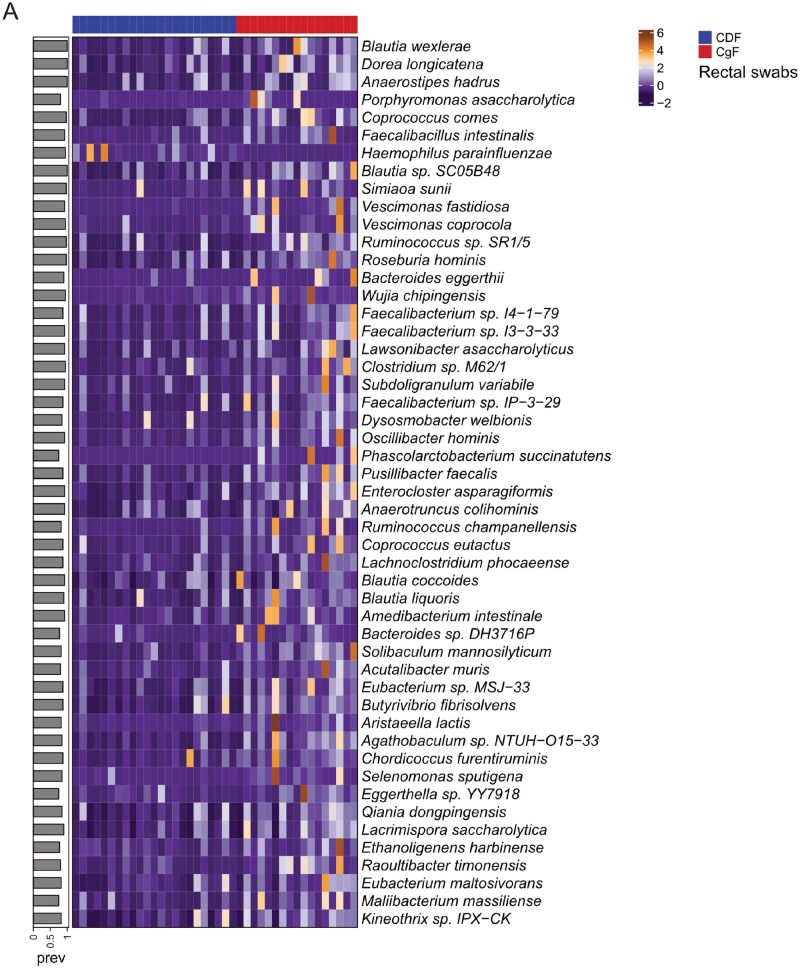
The bacterial composition within CD fistula tracts is representative of a dysbiotic microbiome on a species level. Heatmap of the top 30 bacterial species significantly different in relative abundance in CDF and CgF in the rectal swabs of each patient (A) and in curettage material of each fistula tract (B). Each column represents a sample, here clustered by disease origin. Graphical representations of the relative species abundance in rectal swabs (C) and in fistula tracts (D) of CD or Cg origin.

Next, we investigated the individual bacterial composition at the species level within the communities. In the rectal swabs, we identified overall 951 bacterial species that were found at different frequencies in patients with CDF and CgF. The top 50 of those species are listed in the heatmap in Figure 5A. In contrast, only 37 bacterial species differed in abundance between CDF and CgF in the curettage material (Figure 5B). When comparing the most abundant bacterial species detected in the rectal swabs, we found that patients with CDF exhibited particularly high levels of *Enterococcus* ssp. compared to patients with CgF, while potentially beneficial species such as *Blautia wexlerae* or *Anaerostipes hadrus* were reduced (Figure 5C). In contrast, we detected mainly pathogenic bacterial species in the fistula tracts (Figure 5D). In particular, the fistula tracts of patients with CDF displayed an overrepresentation of *Phocaecola* and *Bacteroides* ssp. when compared to the fistula tracts of patients with CgF. Interestingly, we also found a considerable abundance of *Staphylococcus aureus* in the fistula tracts of patients with CD, while this species was almost absent in the tracts of patients with CgF. Furthermore, the potentially pathogenic bacteria *Fusobacterium animalis* and *Parvimonas micras* were most abundant in the fistula tracts of patients with CgF, while they were almost absent in CDF. These data show that potentially pathogenic species accumulate in the fistulas, which may contribute to their persistent inflammation.

### Serum metabolomics reveal subtle differences between patients with CDF and CgF

**Figure 6.**
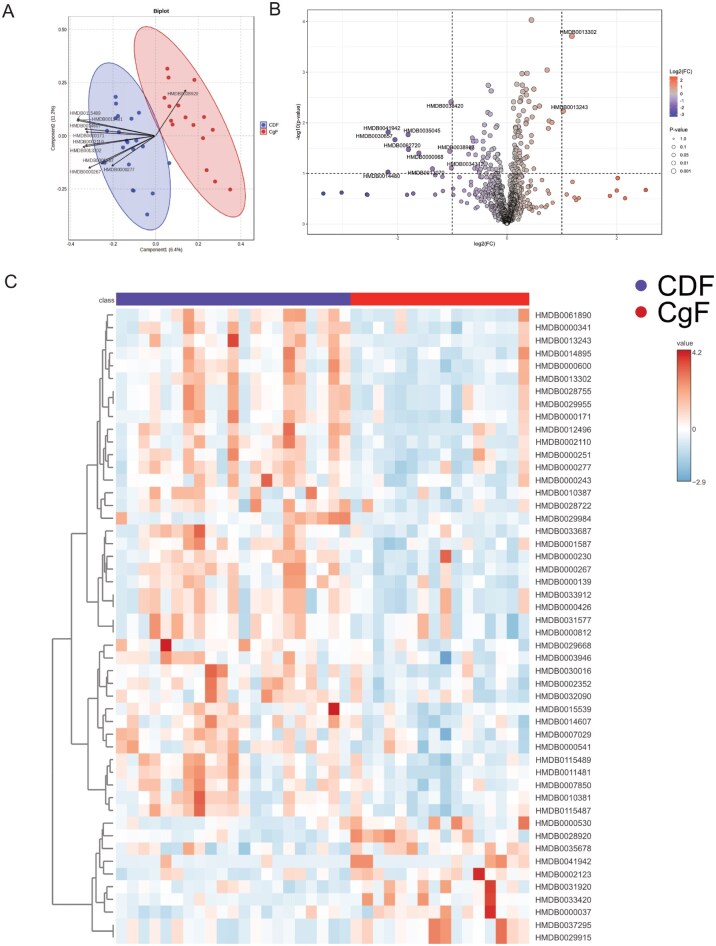
The serum metabolome compositions of patients with CD-associated fistula (CDF) and patients with fistula of cryptoglandular origin (CgF) demonstrate moderate differences. (A) Metabolomics analysis with supervised dimensionality reduction method (partial least-squares discriminant analysis, PLS-DA) indicates the separation between CDF and CgF metabolite profiles. Each dot represents the serum of one patient at intervention. (B) Volcano plot of different abundant metabolites in CDF and CgF (fold change). (C) Heatmap of the 50 metabolites most abundant in serum samples from patients with CgF and CDF. Each column represents a patient’s serum. Each line represents a metabolite. Supervised clustering according to disease origin highlights a metabolic profile of both groups here without significant differences (CDF = 23, CgF = 17).

To elucidate the differences between patients with CDF and CgF at a systemic level we investigated the metabolic profile in the serum of patients with fistulizing disease. Partial least-squares discriminant analysis (PLS-DA) revealed two distinct clusters representative of patients with CDF and CgF (Figure 6A), whereas the unsupervised PCA indicated no difference (Figure S11). Statistical analysis did not reveal any significant differences between the two groups regarding serum metabolites. Nevertheless, the metabolites differing most strongly were HMDB0033420 (gibberellin A55), HMDB0041942 (*N*-acetyl-*S-(N*-methylcarbamoyl)cysteine), HMDB0035045 (gibberellin A39), HMDB0030657 (carinol), HMDB0062720 (thymol sulfate), HMDB0000068 (epinephrine), and HMDB0038967 (1-propenyl 1-(1-propenylthio)propyl disulfide), which were most abundant in the serum of patients with CDF. In contrast, HMDB0013302 (phenylalanylphenylalanine) and HMDB0013243 (leucylphenylalanine) were most abundant in the serum of patients with CgF (Figure 6B). The 50 most abundant metabolites identified in serum of our patients are listed in the heatmap (Figure 6C). Overall, we found rather modest differences in the serum metabolome between patients with CDF or CgF.

### Circulating cytokine analysis by multiplex-ELISA

**Figure 7.**
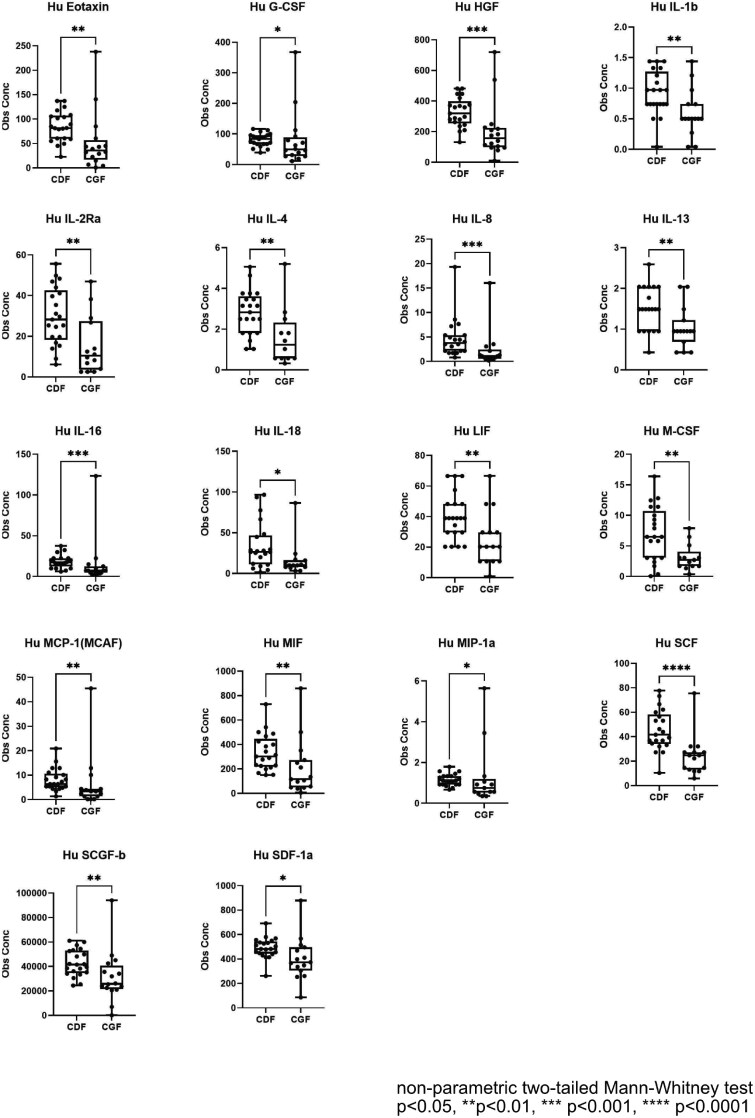
Serum cytokines of patients with CD-associated fistula (CDF) reveal signs of chronic inflammation when compared to patients with fistula of cryptoglandular origin (CgF). Graphical representation of observed concentration of 23 cytokines significantly different (out of 40 measured) in the serum of CDF (*n* = 23) compared to serum of CgF (*n* = 17) patients. The number of samples measured for each cytokine is indicated in Table S2 (box with 25th and 75th percentiles, whiskers with minimum to maximum values, all points shown; two-tailed Mann–Whitney U test, *P*-values: *P < .05, **P < .01, ***P < .001, ****P < .0001). Data are shown as boxplots with median, interquartile range, and 1.5 × IQR whiskers; individual observations are overlaid with jitter.

We next investigated the serum cytokine profile in patients with either CgF or CDF by multiplex-ELISA. In the serum of patients with CD, we found a higher abundance particularly of chemoattractant molecules (such as eotaxin or MCP-1) as well as of cytokines that had previously been described in connection with CDF (such as IL-1b, IL-4, IL-8, IL-13 or IL-18) (Figure 7). Of note, TNF levels were not significantly different and IL-1α, IL-1ra, IL-17, and various interferons (IFN-α and IFN-γ) also showed similar levels in both groups (Table S2).

## Discussion

Perianal fistulas remain a significant challenge in patient care, and particularly complex fistulas are difficult to treat, while they have a tremendous impact on patients’ quality of life. In this study, we performed multiomics analysis of tissues and serum derived from patients with perianal CDF or CgF. Overall, the transcriptomes observed in the rectal biopsies (located 2–3 cm from the internal opening of the fistula) were clearly different from the transcriptomes found in the curettage materials derived from the fistula tracts, regardless of the underlying condition (CDF or CgF). When comparing mucosal rectal biopsies between both disease etiologies, we observed significant differences particularly regarding the inflammatory status, which appeared to be elevated in patients with CD. Interestingly, processes related to the development of the epidermis, keratinization, and keratinocyte differentiation were also upregulated in the mucosal biopsies of patients with CD compared to patients with CgF. This is well in line with data recently published by Becker et al. that showed similar results in bulk RNA-seq analysis when comparing CD-related internal fistula openings to normal rectal mucosa.^41^ Luminal disease activity was absent in our study cohort, since the absence of active luminal activity was a required criterion for treatment. In the work of Gudino et al., single cell-based transcriptomics analysis of rectal biopsies of a stratified CD cohort indicated a signature associated with fistula formation, but this was independent of the presence or absence of active rectal inflammation.^42^ This result suggests that our findings with CDF patients in luminal remission might indeed be representative also for patients with CDF with luminal inflammation. Finally, the differences observed in our study in the rectal mucosa of patients with CgF and patients with CD may be interpreted as the underlying transcriptomic inflammatory signature of CD, or as reminiscent processes after healing of rectal mucosal lesions.

Interestingly, the transcriptome data from curettage material of CDF and CgF origin were quite similar as only a few significant differences were detected. Genes specific to perianal fistulas and common in patients with CDF and CgF were related to EMT, inflammatory response and TNF-signaling via NF-κB. These processes play a critical role in wound healing and tissue regeneration. Furthermore, by focussing on the common DEGs between CDF and CgF found in biopsies and fistulas we identified a panel of 18 genes. Within a single rectal biopsy, this panel could discriminate patients with CgF from those with CDF. Such a diagnostic tool (eg, combined with the measurement of fecal calprotectin) could help to distinguish between patients with perianal fistulas as the first manifestation of CD and patients with fistulas of cryptoglandular origin, thereby enabling adequate and early anti-inflammatory treatment.^43^

On the metagenomics level, the intestinal microbiome of patients with fistulizing CD is representative of the typical dysbiosis found in IBD patients (even if the CD patients are mostly in luminal remission).^44^ The loss of compositional diversity and the increase in pathogenic species such as *Enterococci* ssp. was also observed in fistulas of patients with CD. Interestingly, beneficial species (with anti-inflammatory properties such as *Bifidobacterium longum*) were detected in rectal biopsies of patients with CgF and CDF. While we identified rectal microbiome alterations in CDF, we acknowledge as a limitation of our study that we were not able to definitively isolate the fistulizing-specific microbial signature from the broader baseline dysbiosis observed in CD. Future standardized, multi-center studies with closely matched, non-fistulizing CD control cohorts might be needed to disentangle these anatomical-specific features.

Finally, the serum metabolome did not indicate strong differences between patients with CD and non-IBD patients on the day of intervention. The expected difference in serum metabolites between IBD and non-IBD patients might be missing because the patients with CD included in this study were in luminal remission. However, the circulating cytokine profiles indicated an elevation of some pro-inflammatory mediators such as IL-4 and IL-13 (cytokines relevant for Th2 signature) in patients with CDF compared to patients with CgF.^45^ Interestingly, the Th2 signature was also found to be elevated in the transcriptome of rectal biopsies of CDF compared to CgF patients. However, this difference was absent when comparing the curettage transcriptome between CDF and CgF (Figure S12).

Our results obtained from transcriptomic data reveal that the molecular makeup of established perianal fistulas is quite similar in patients with CDF and CgF, whereas it differs strikingly within the rectal biopsies (located 2–3 cm from the internal openings): the main differences in the rectal biopsies are related to immune and inflammatory responses as well as to programs in epidermal development and keratinocyte differentiation. Previous publications identifying epithelium in perianal fistula suggested that epithelialization in the fistula tract might prevent the fistula from closing.^46^^,^^47^ In contrast, Becker et al. showed that their new fistula keratinization score correlates inversely with fistula disease severity and disease outcome.^48^ They also showed that wound healing activity is associated with the development of squamous epithelium and finally that this activity is more pronounced in CgF than in CDF. We might hypothesize that normal wound healing in perianal fistulas consists in the replacement of the granulation tissue-lined lumen with a stratified squamous epithelium. Therefore, a complementary approach for fistula healing in the context of CD might be to support development of squamous epithelium in the fistula tracts (but without inducing dysplasia).

Our metagenomic results showed an accumulation of mainly potential pathogenic bacterial species in the fistula tracts compared to the rectal microbiome. Selecting and applying specific antibiotics (or in the future perhaps specific bacteriophages) targeting pathogenic bacterial species in the fistula tracts in CDF and CgF might improve fistula healing. Additional approaches to modulate the fistula microbiome (eg, by probiotics) might also help to replace the pathogenic population.

Our study has several limitations as the number of patients included is rather small (23 CD vs 17 CgF). Small group sizes limit power and complicate interpretation of subgroup differences. Patients with CD generally have different medical histories, which include treatment regimens, disease duration, timepoint of fistula formation, recurrence, and lack of response to treatments. These variables enhance the heterogeneity of the CD group. Also, in relatively small, specialized surgical cohorts, random sampling variation can lead to anatomical distributions that do not perfectly mirror global prevalence rates. Consequently, we are limited in our ability to perform robust stratified analyses (eg, comparing distinct anatomical subtypes within or across disease groups), as the statistical power within these sparse anatomical subcategories would be highly insufficient and prone to Type II errors. Furthermore, analyzing the results regarding fistula relapse and healing after intervention is only feasible with large cohorts (eg, to identify biomarkers). On the molecular level, expression of proteins involved in the pathways observed needs to be confirmed.

Nevertheless, we propose here a panel of transcripts to differentiate the etiology of perianal fistulas, enabling proper treatment. In particularly in patients with CD, the pretreatment could postpone luminal inflammatory activation.^39^ Determining the optimal combination of different medical and surgical treatment methods for the various types of fistulas might be a step toward improving healing—in addition to a better understanding of the molecular mechanisms underlying the triggering, development, and maintenance of fistulas.

## Conclusion

Our study on patients with perianal fistulas shows that fistula tissues are quite similar on a transcriptomic level, regardless of the underlying cause of the fistula. In contrast, the microbiome residing in the fistula tracts are representative of the bacterial population found in the rectum, signifying that the dysbiosis in patients with CD was also found in the fistula microbiome. However, we detected a significant enrichment of pathogenic, albeit different, species in the tracts of patients with either CDF or CgF. In summary, our data suggest that, at a molecular level, the fistula tracts of CDF and CgF patients are quite comparable and, therefore, the trigger inducing fistula formation and the molecular mechanisms enabling its expansion and maintenance may also be similar.

## Supplementary Material

jjag080_Supplementary_Data

## Data Availability

The data underlying this article cannot be shared publicly due to the privacy of individuals who participated in the study.
