# Supplementary material for: Multiomics analysis dissects the molecular foundation of perianal fistulas associated with Crohn’s disease and of cryptoglandular origin
Source: J Crohns Colitis. 2026 Jun 30;20(6):jjag080. doi: 10.1093/ecco-jcc/jjag080 (PMC13318227; doi:10.1093/ecco-jcc/jjag080)
Supplement: jjag080_Supplementary_Data [file jjag080_supplementary_data.zip › Mamie_FISTULA_Supplemental_material_20260508.docx]

**SUPPLEMENTAL MATERIAL**

**Methods for multiomics analyses**

**RNA sequencing**

Total RNA expression in fistula (curettage material) and rectal mucosa (biopsies) located near the internal opening of the fistula (within 2-3 cm) was analyzed by bulk-RNA sequencing in collaboration with the Functional Genomics Center Zurich (FGCZ), Switzerland. After thawing and homogenizing the collected tissues with gentleMACS™ Dissociator (Miltenyi Biotec, US), RNA isolation was quickly performed with Maxwell RSX simply RNA Tissue Kit (cat. AS1340, Promega, US) according to the manufacturer’s instructions. FGCZ controlled RNA quality by capillary electrophoresis. In addition, library preparations and high throughput transcriptome sequencing were performed with Illumina Novaseq 6000, full S1 Flowcell and Truseq mRNA protocol with paired end (150 bp) read configuration. The human genome GRCh38 from Ensemble was used as a reference genome for STAR (V.2.5.4) for mapping the reads. The use of technical replicates and quality control analysis with FastQC (V.0.12.0, Babraham Institute, UK) indicated no batch effect and no cohort effect. 87 samples were used for further analysis. Cell type deconvolution was performed using the immunedeconv R package with the quanTIseq algorithm, which estimates the proportions of immune cell types from bulk transcriptomic profiles [^1^](#_ENREF_1)^,^[^2^](#_ENREF_2).

**Differential Gene Expression (DEG)**

Differential gene expression (DEG) analysis of experimental groups was performed using the Bioconductor package DESeq2. Each subgroup (biopsies-CgF, biopsies-CDF, curettage-CgF, curettage-CDF) was taken as a single factor. The genes were selected based on the following comparisons: for volcano-plots and heatmaps, biopsies-CDF to biopsies-CgF, curettage-CDF to curettage-CgF (DEG levels were considered significant with adjusted p-value ≤0.05). The Database for Annotation, Visualization, and Integrated Discovery (DAVID) was used for gene ontology analysis [^3^](#_ENREF_3)^,^[^4^](#_ENREF_4). For the common genes found in CDFs and CgFs, comparisons of biopsies vs curettage of CDF and of biopsies vs curettage of CgF were made (adjusted p-value ≤ 0.001 and log2FC ≤-2 for upregulated genes in curettage, log2FC ≥2 for upregulated genes in biopsies). The genes common among the upregulated genes in curettage compared to biopsies, and common to CDF and CgF (and similarly for the downregulated genes) were identified using Venn diagram. Enrichr was used for pathways analysis in curettage and to determine the cell composition in biopsies [^5^](#_ENREF_5)^,^[^6^](#_ENREF_6).

**Metagenomics**

In a pilot study, feces and rectal swabs from same patients were analyzed by metagenomics analysis and showed similar bacterial profiles (data not shown). Rectal swabs and fistula curettage material from fistulizing patients were analyzed by Shotgun metagenomics analysis in collaboration with Microsynth, Balgach, Switzerland. DNA was isolated, fluorescence-based quantification of dsDNA and an integrity check of the DNA by capillary electrophoresis was performed. TruSeq libraries were sequenced on an Illumina NovaSeq S2 flow cell, 2*100. This sequencing platform achieved 30 million reads per sample on average. After quality check and adaptor trimming using fastp [^7^](#_ENREF_7), human reads were removed by mapping to the GRCh38 human genome using bowtie2 [^8^](#_ENREF_8). Rarefaction was applied using 100,000 reads to unify the depth before diversity calculations. Alpha- and beta-diversity were calculated using alpha and ordinate functions in the microbiome and phyloseq R packages. Taxonomic classification was performed using Kraken2 against the PlusPF database with the default parameters [^9^](#_ENREF_9). Only bacterial counts were used for the calculation of relative abundance. Functional annotation was conducted using HUMAnN3 by mapping against the UniRef90 database [^10^](#_ENREF_10). The average matched results from forward and reverse reads were used for downstream analysis. Statistical analyses were performed in R (version 4.4.3). Permutational multivariate analysis of variance (PERMANOVA) was conducted using the adonis2 function in vegan to assess differences in community composition between groups. Differential abundance was assessed using relative abundance data via a two-way ANOVA, followed by pairwise Wilcoxon rank-sum post-hoc tests. Resulting p-values were adjusted for multiple comparisons using the false discovery rate (FDR) method.

**Metabolomics**

After blood withdrawal in BD Vacutainer™ (cat. 367896, BD, Switzerland) and a latency of at least 20 minutes, serum was isolated from coagulated blood [^11^](#_ENREF_11) and extracted by methanol precipitation. Metabolomics analysis was carried out by flow-injection mass spectrometry on Agilent QTOF-6550 as described previously by Fuhrer et al. [^12^](#_ENREF_12). The electrospray ionization was performed in both positive and negative modes. In positive ionization mode, the mobile phase was methanol/water (60:40, v/v) with 0.1% formic acid (pH 3). In negative mode, the mobile phase consisted of isopropanol/water (60:40, v/v) buffered with 5 mM ammonium carbonate (pH 9). The flow rate was maintained at 150 μL/min, with sample injection volume of 1.6 μL in full-loop mode. Metabolites were annotated based on accurate mass and isotopic patterns. A total number of 1854 metabolites were putatively identified in the 40 serum samples. We used MetaboAnalystR package version 4.0.0 for the statistical analysis.

**Cytokines analysis by Multiplex-ELISA**

Relative cytokine concentrations in the blood serum were analyzed by Multiplex-ELISA. Serum samples were diluted 1/4 and used with Pro-Human-Cytokine-Screening-Panel, 48-Plex kit (cat. 12007283, BioRad, US) run on Luminex device as instructed. For statistical analysis, two-tailed Kruskal-Wallis followed by Pairwise Wilcoxon Rank-Sum Test were performed using GraphPad Prism v10.5.0 for Windows, GraphPad Software, Boston, USA. Exact p-values and definition of n (including extrapolated values) are listed for each cytokine (Table S2).

**REFERENCES (SUPPLEMENTAL MATERIAL)**

1. Sturm G, Finotello F, List M. Immunedeconv: An R Package for Unified Access to Computational Methods for Estimating Immune Cell Fractions from Bulk RNA-Sequencing Data. Methods Mol Biol. 2020;2120:223-32.

2. Finotello F, Mayer C, Plattner C, Laschober G, Rieder D, Hackl H, Krogsdam A, Loncova Z, Posch W, Wilflingseder D, Sopper S, Ijsselsteijn M, Brouwer TP, Johnson D, Xu Y, Wang Y, Sanders ME, Estrada MV, Ericsson-Gonzalez P, Charoentong P, Balko J, de Miranda N, Trajanoski Z. Molecular and pharmacological modulators of the tumor immune contexture revealed by deconvolution of RNA-seq data. Genome Med. 2019;11(1):34.

3. Sherman BT, Hao M, Qiu J, Jiao X, Baseler MW, Lane HC, Imamichi T, Chang W. DAVID: a web server for functional enrichment analysis and functional annotation of gene lists (2021 update). Nucleic Acids Res. 2022;50(W1):W216-W21.

4. Huang da W, Sherman BT, Lempicki RA. Systematic and integrative analysis of large gene lists using DAVID bioinformatics resources. Nat Protoc. 2009;4(1):44-57.

5. Xie Z, Bailey A, Kuleshov MV, Clarke DJB, Evangelista JE, Jenkins SL, Lachmann A, Wojciechowicz ML, Kropiwnicki E, Jagodnik KM, Jeon M, Ma'ayan A. Gene Set Knowledge Discovery with Enrichr. Current Protocols. 2021;1(3):e90.

6. Kuleshov MV, Jones MR, Rouillard AD, Fernandez NF, Duan Q, Wang Z, Koplev S, Jenkins SL, Jagodnik KM, Lachmann A, McDermott MG, Monteiro CD, Gundersen GW, Ma'ayan A. Enrichr: a comprehensive gene set enrichment analysis web server 2016 update. Nucleic Acids Res. 2016;44(W1):W90-7.

7. Chen S, Zhou Y, Chen Y, Gu J. fastp: an ultra-fast all-in-one FASTQ preprocessor. Bioinformatics. 2018;34(17):i884-i90.

8. Langmead B, Wilks C, Antonescu V, Charles R. Scaling read aligners to hundreds of threads on general-purpose processors. Bioinformatics. 2019;35(3):421-32.

9. Wood DE, Lu J, Langmead B. Improved metagenomic analysis with Kraken 2. Genome Biol. 2019;20(1):257.

10. Beghini F, McIver LJ, Blanco-Míguez A, Dubois L, Asnicar F, Maharjan S, Mailyan A, Manghi P, Scholz M, Thomas AM, Valles-Colomer M, Weingart G, Zhang Y, Zolfo M, Huttenhower C, Franzosa EA, Segata N. Integrating taxonomic, functional, and strain-level profiling of diverse microbial communities with bioBakery 3. Elife. 2021;10.

11. Morsy Y, Walberg A, Wawrzyniak P, Hubeli B, Truscello L, Mamie C, Niechcial A, Gueguen E, Manzini R, Gottier C, Lang S, Scharl S, Blumel S, Biedermann L, Rogler G, Turina M, Ramser M, Petrowsky H, Arnold IC, Zeissig S, Zamboni N, Egli A, Niess JH, Hruz P, Knuth A, Fritsch R, Manz MG, Wawrzyniak M, Scharl M. Blood-borne immune cells carry low biomass DNA remnants of microbes in patients with colorectal cancer or inflammatory bowel disease. Gut Microbes. 2025;17(1):2530157.

12. Fuhrer T, Heer D, Begemann B, Zamboni N. High-throughput, accurate mass metabolome profiling of cellular extracts by flow injection-time-of-flight mass spectrometry. Anal Chem. 2011;83(18):7074-80.
