# Supplementary figures and images for: Multiomics analysis dissects the molecular foundation of perianal fistulas associated with Crohn’s disease and of cryptoglandular origin
Source: J Crohns Colitis. 2026 Jun 30;20(6):jjag080. doi: 10.1093/ecco-jcc/jjag080 (PMC13318227; doi:10.1093/ecco-jcc/jjag080)

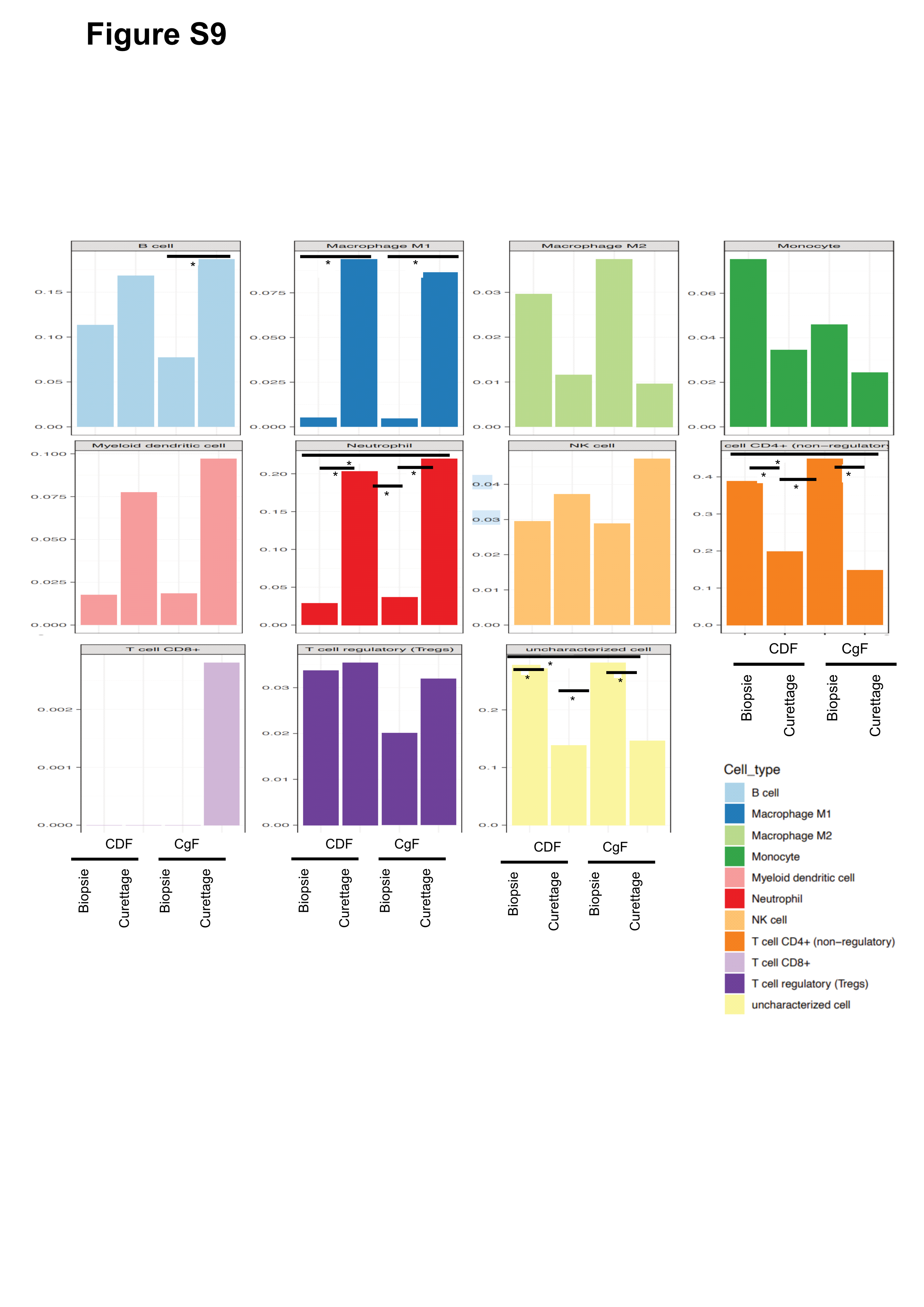

Supplement: jjag080_Supplementary_Data [file jjag080_supplementary_data.zip › Suppl_Fig_Tab_FISTULA_20260508_15.tiff]

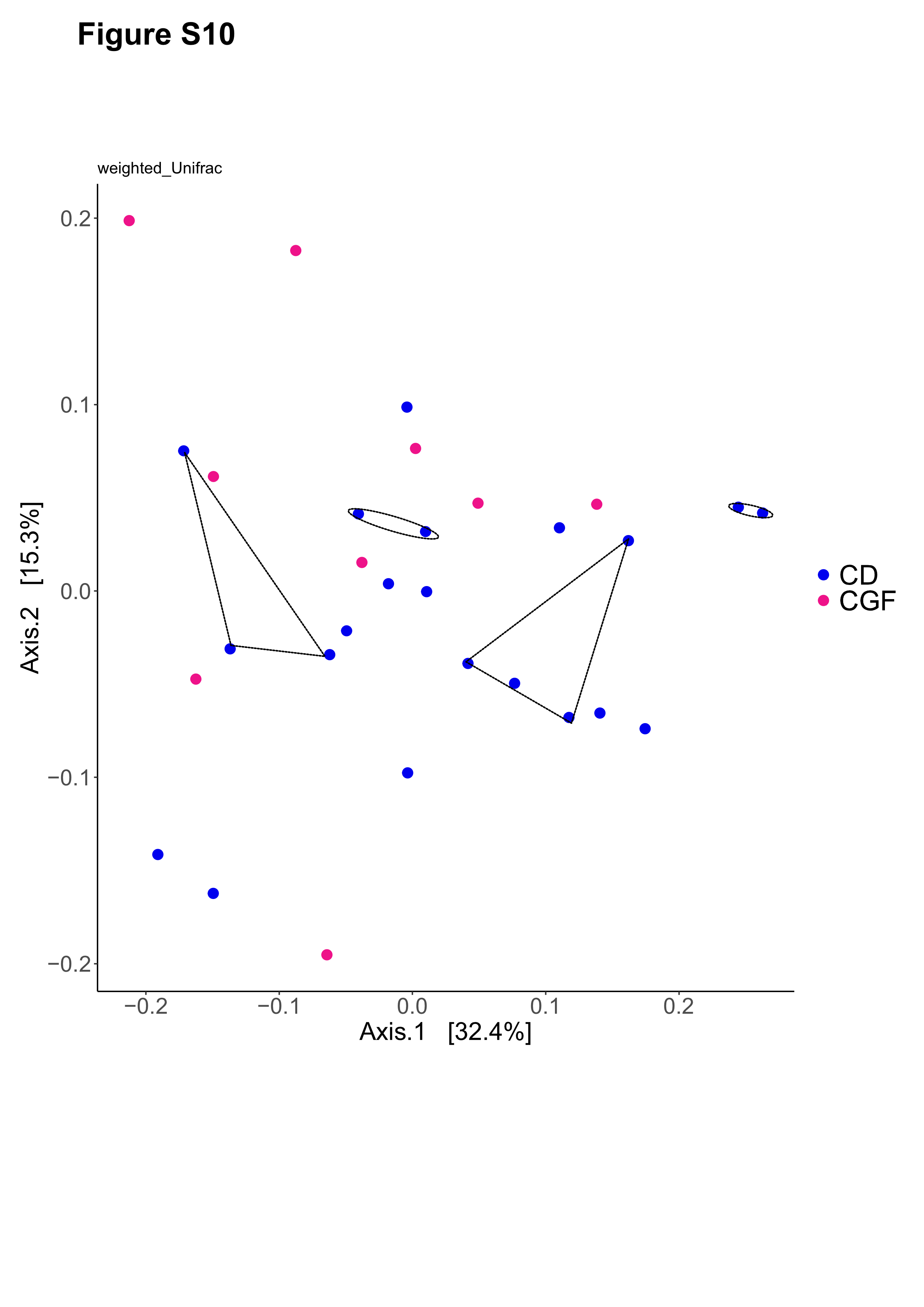

Supplement: jjag080_Supplementary_Data [file jjag080_supplementary_data.zip › Suppl_Fig_Tab_FISTULA_20260508_16.tiff]

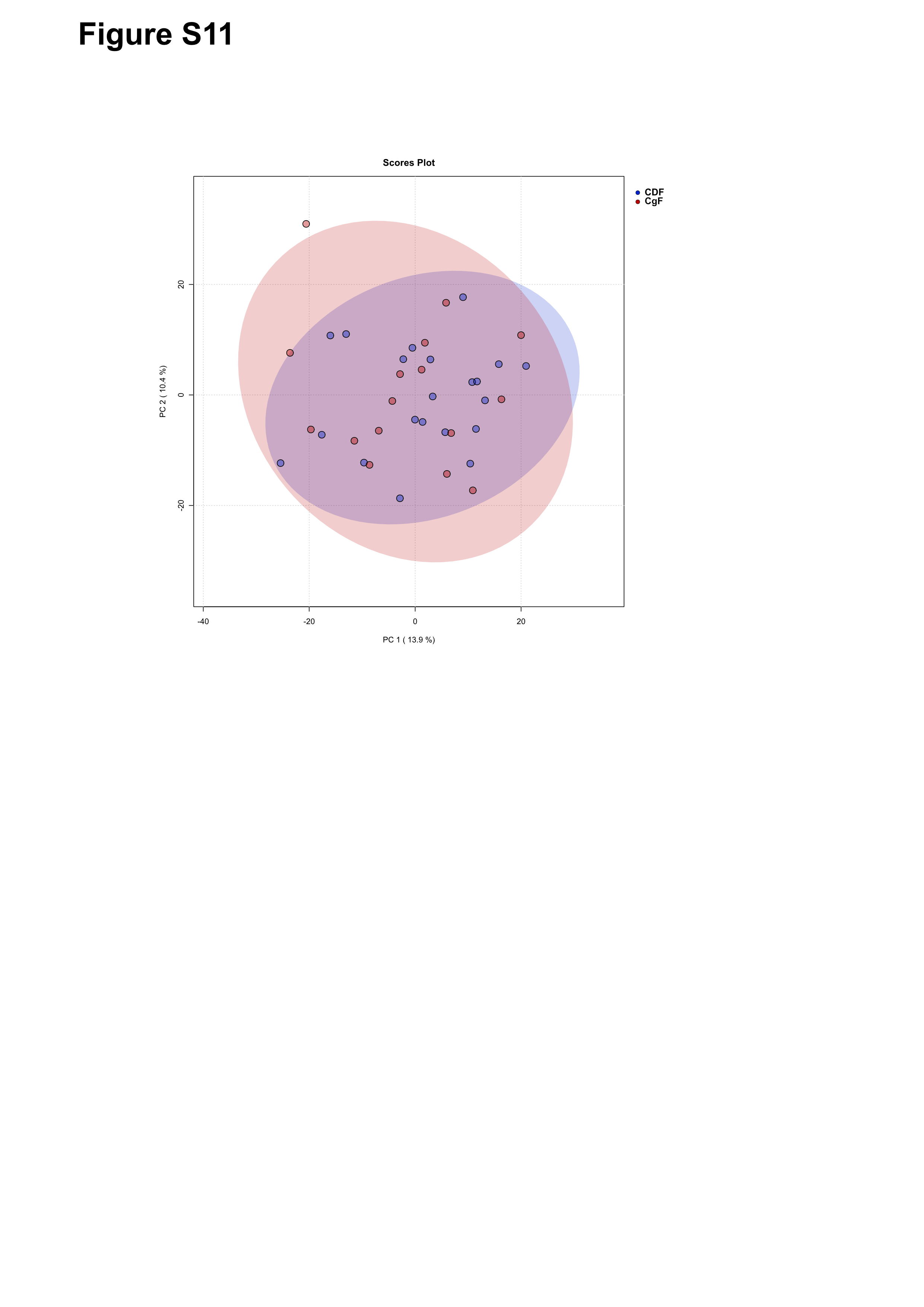

Supplement: jjag080_Supplementary_Data [file jjag080_supplementary_data.zip › Suppl_Fig_Tab_FISTULA_20260508_17.tiff]

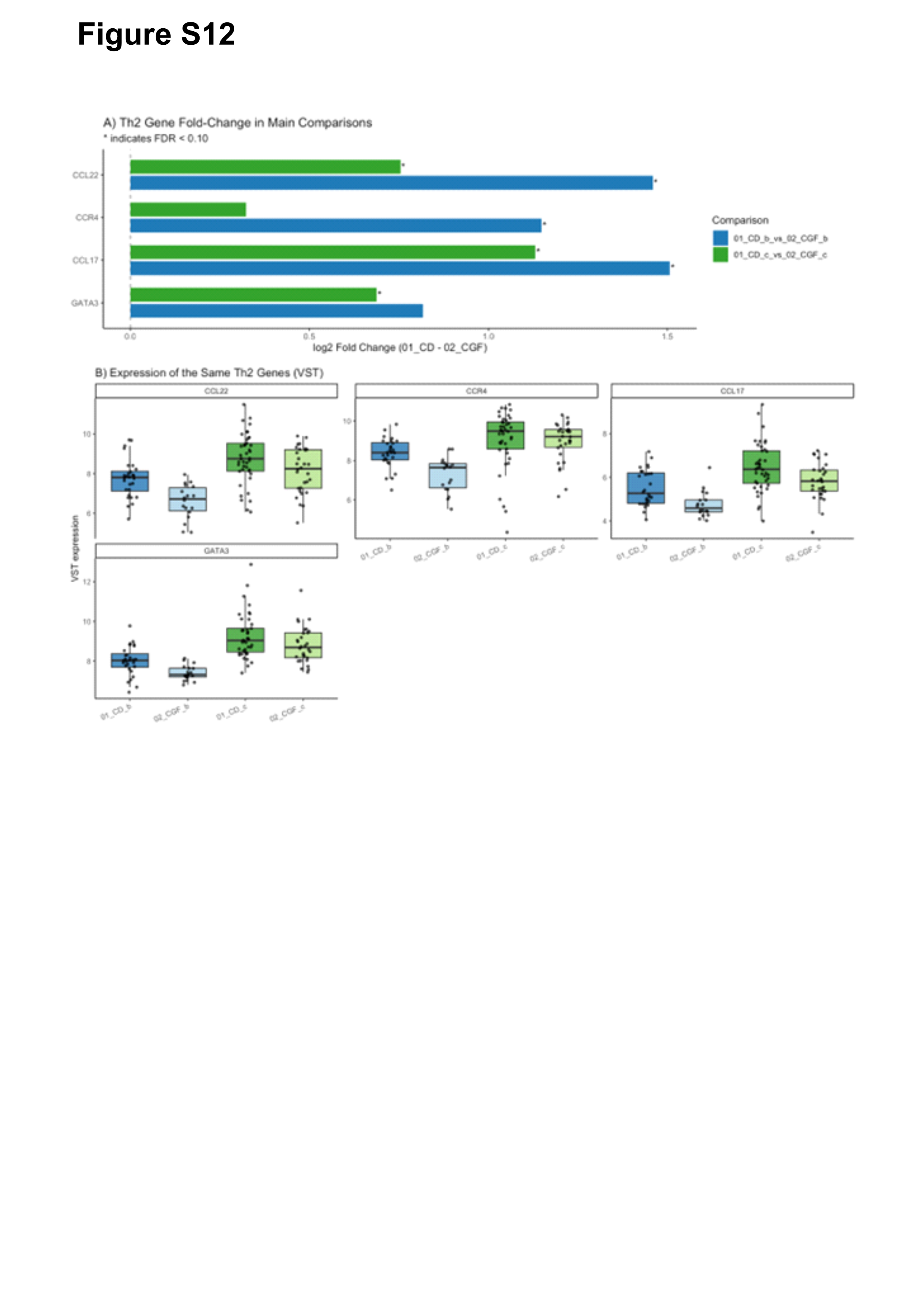

Supplement: jjag080_Supplementary_Data [file jjag080_supplementary_data.zip › Suppl_Fig_Tab_FISTULA_20260508_18.tiff]

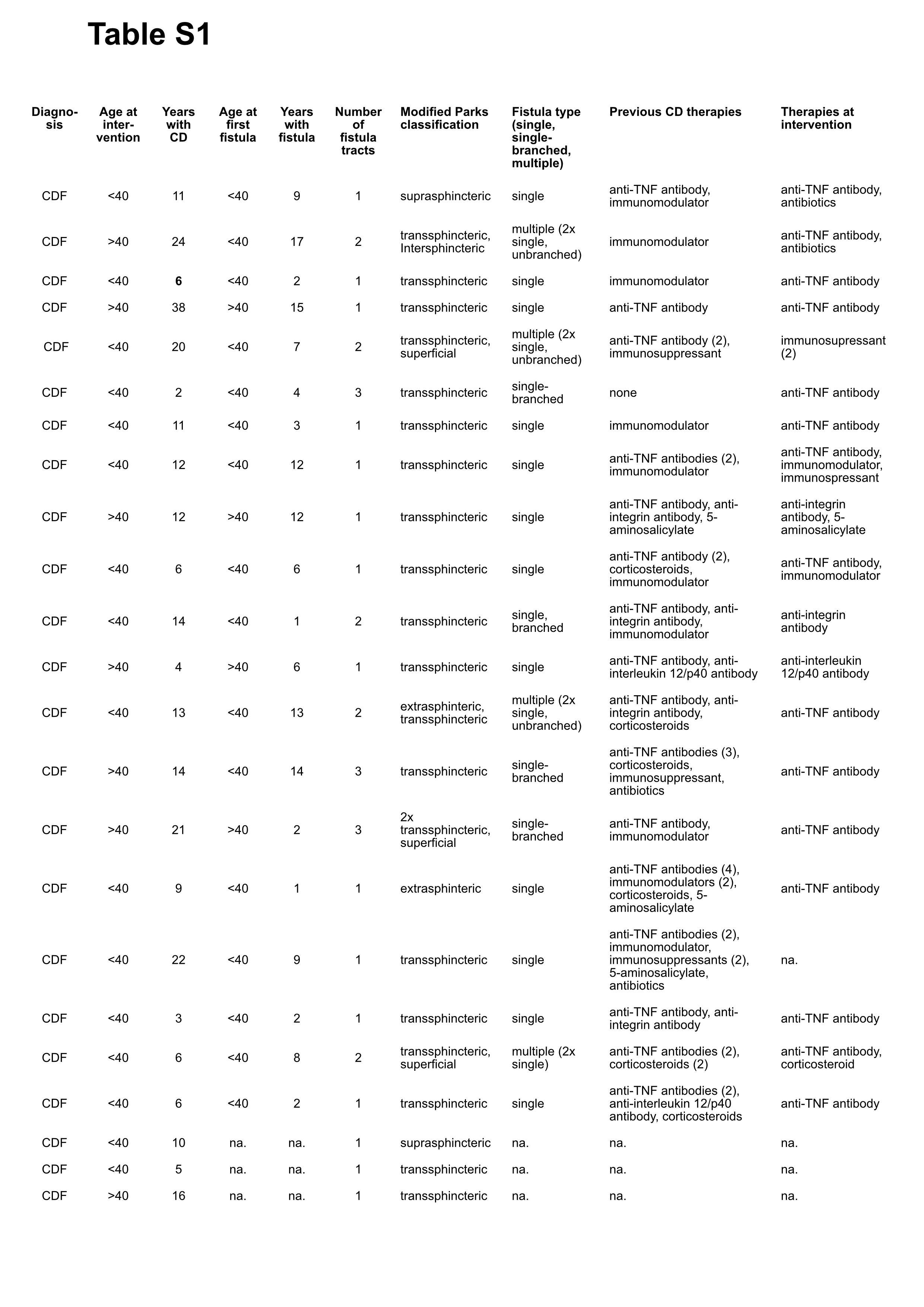

Supplement: jjag080_Supplementary_Data [file jjag080_supplementary_data.zip › Suppl_Fig_Tab_FISTULA_20260508_19.tiff]

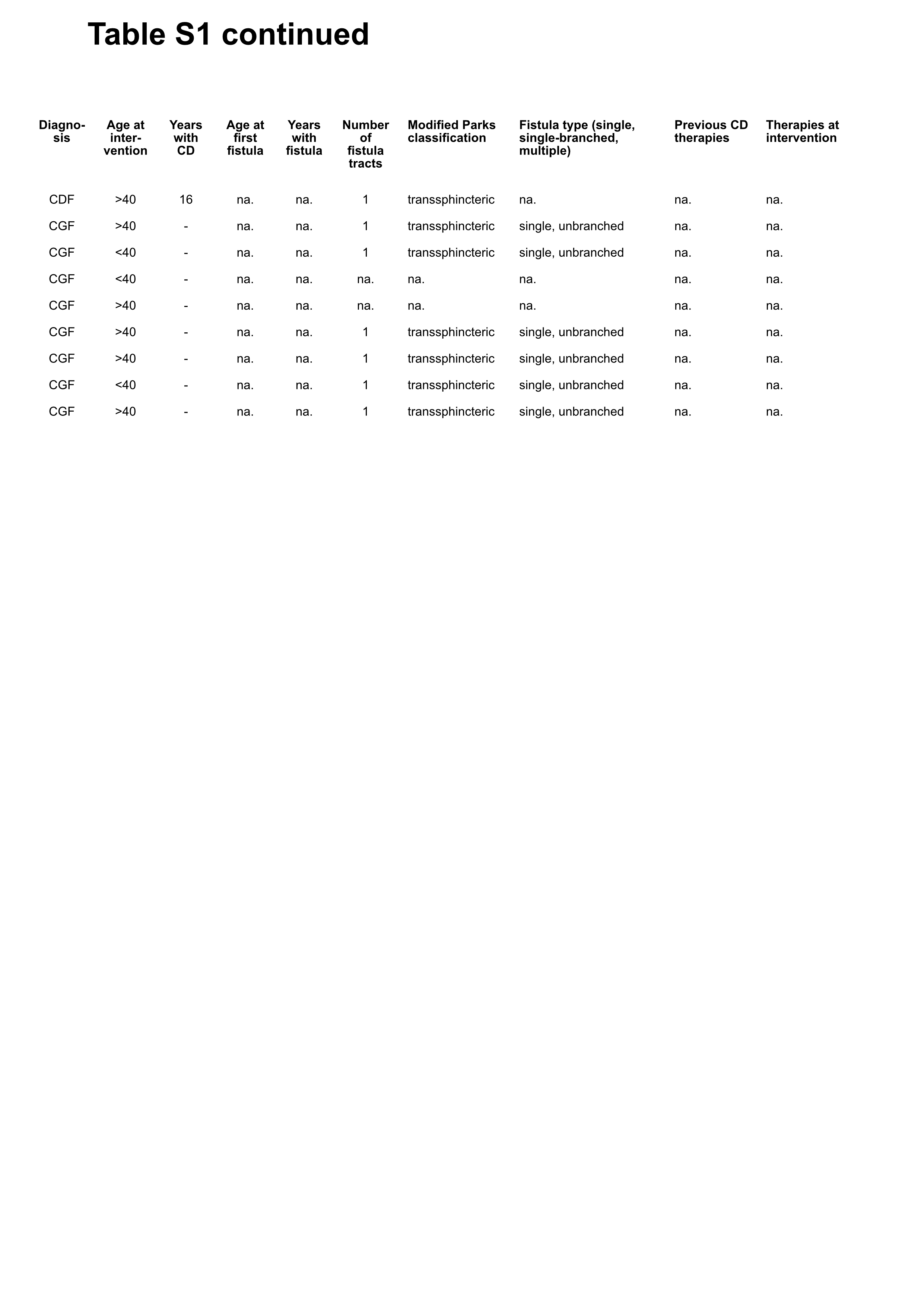

Supplement: jjag080_Supplementary_Data [file jjag080_supplementary_data.zip › Suppl_Fig_Tab_FISTULA_20260508_20.tiff]

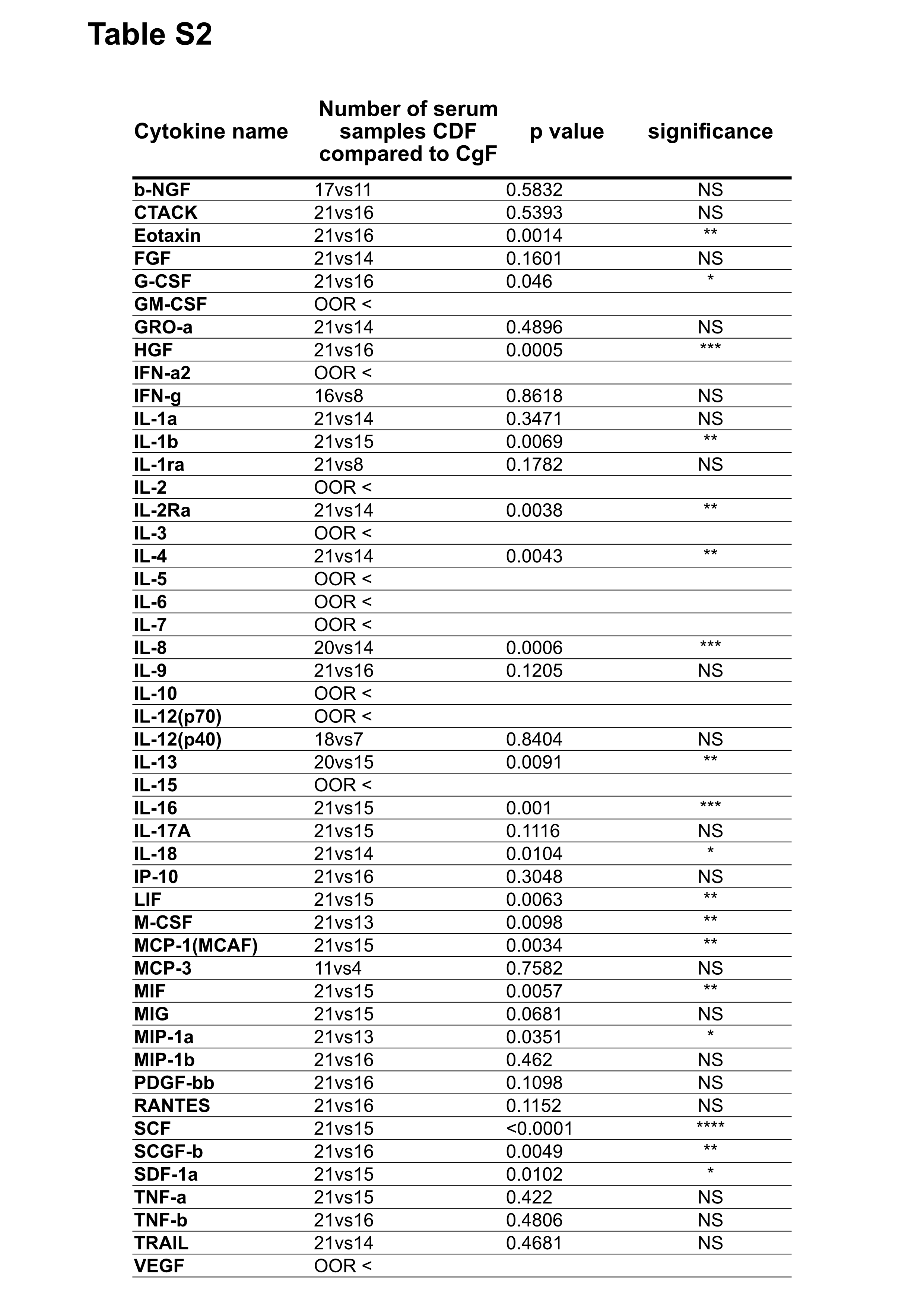

Supplement: jjag080_Supplementary_Data [file jjag080_supplementary_data.zip › Suppl_Fig_Tab_FISTULA_20260508_21.tiff]

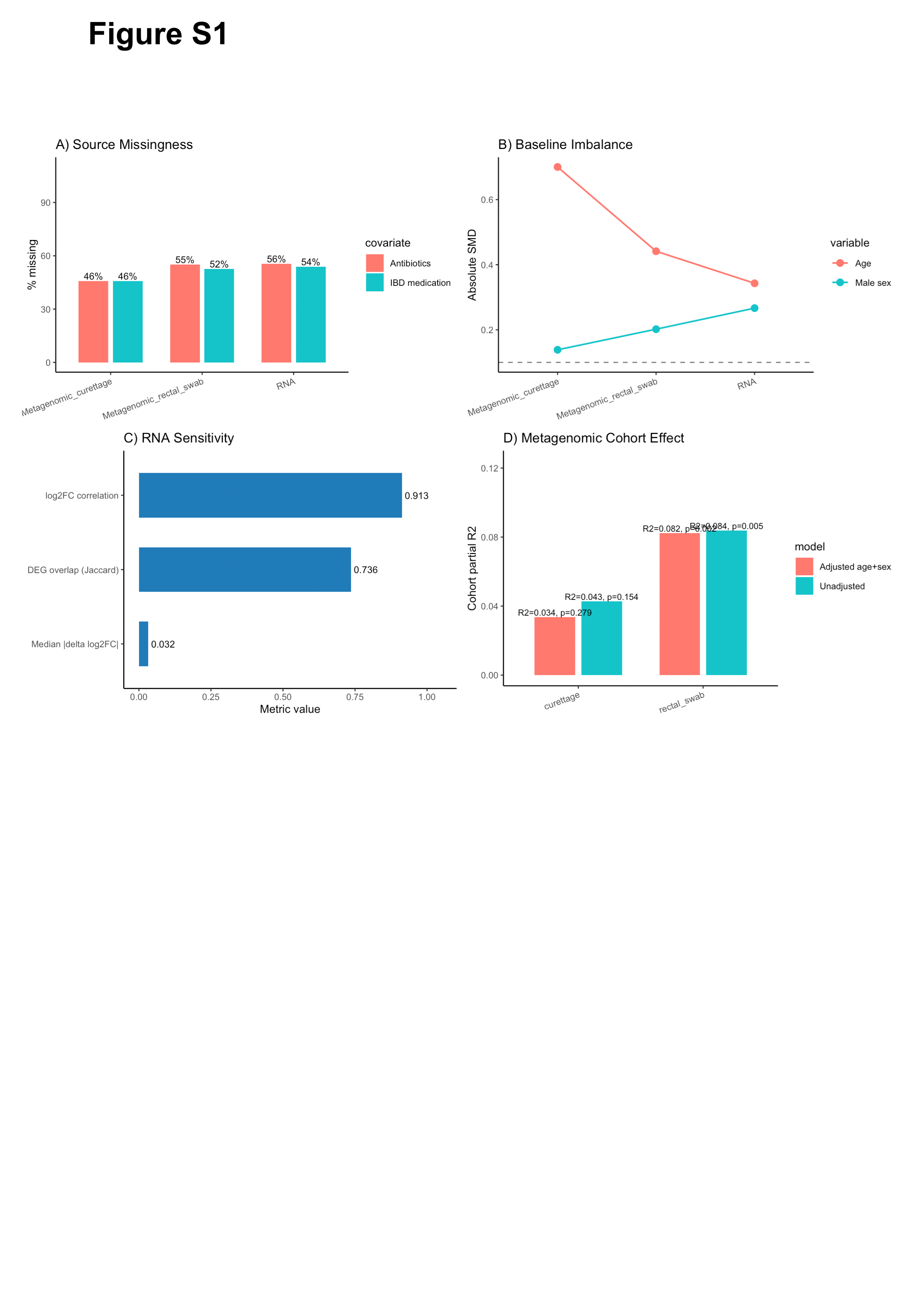

Supplement: jjag080_Supplementary_Data [file jjag080_supplementary_data.zip › Suppl_Fig_Tab_FISTULA_20260508_1.tiff]

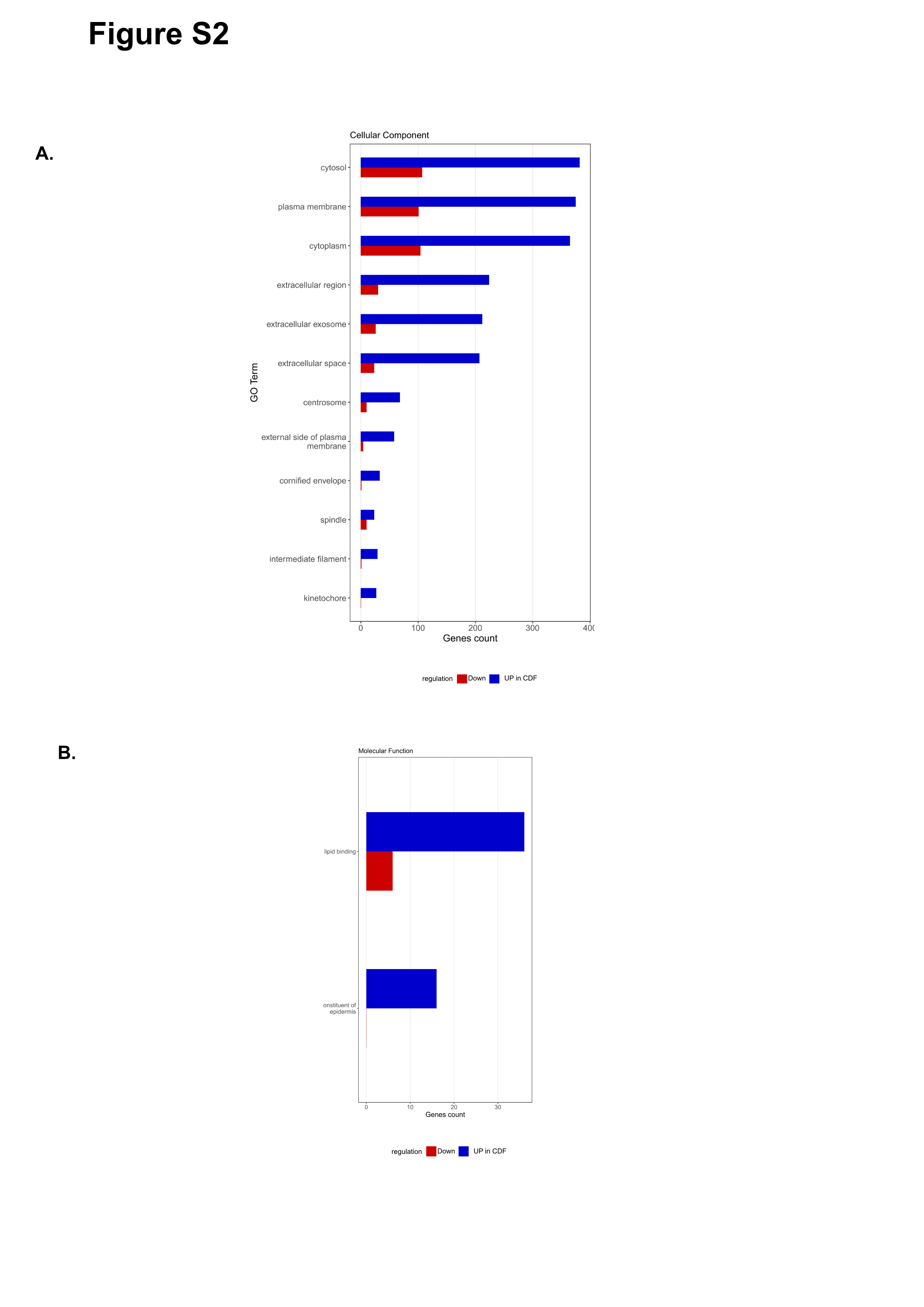

Supplement: jjag080_Supplementary_Data [file jjag080_supplementary_data.zip › Suppl_Fig_Tab_FISTULA_20260508_2.tiff]

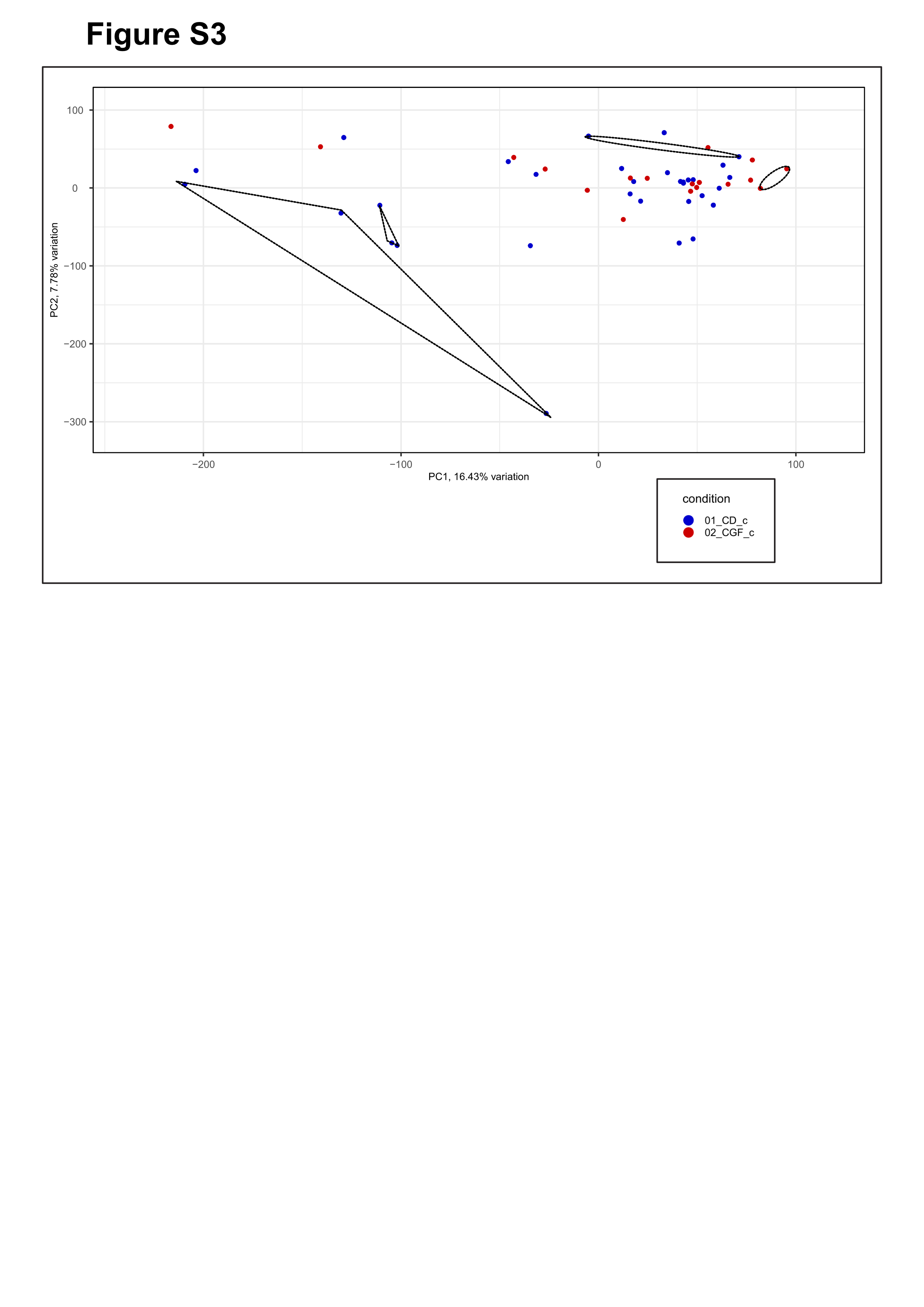

Supplement: jjag080_Supplementary_Data [file jjag080_supplementary_data.zip › Suppl_Fig_Tab_FISTULA_20260508_3.tiff]

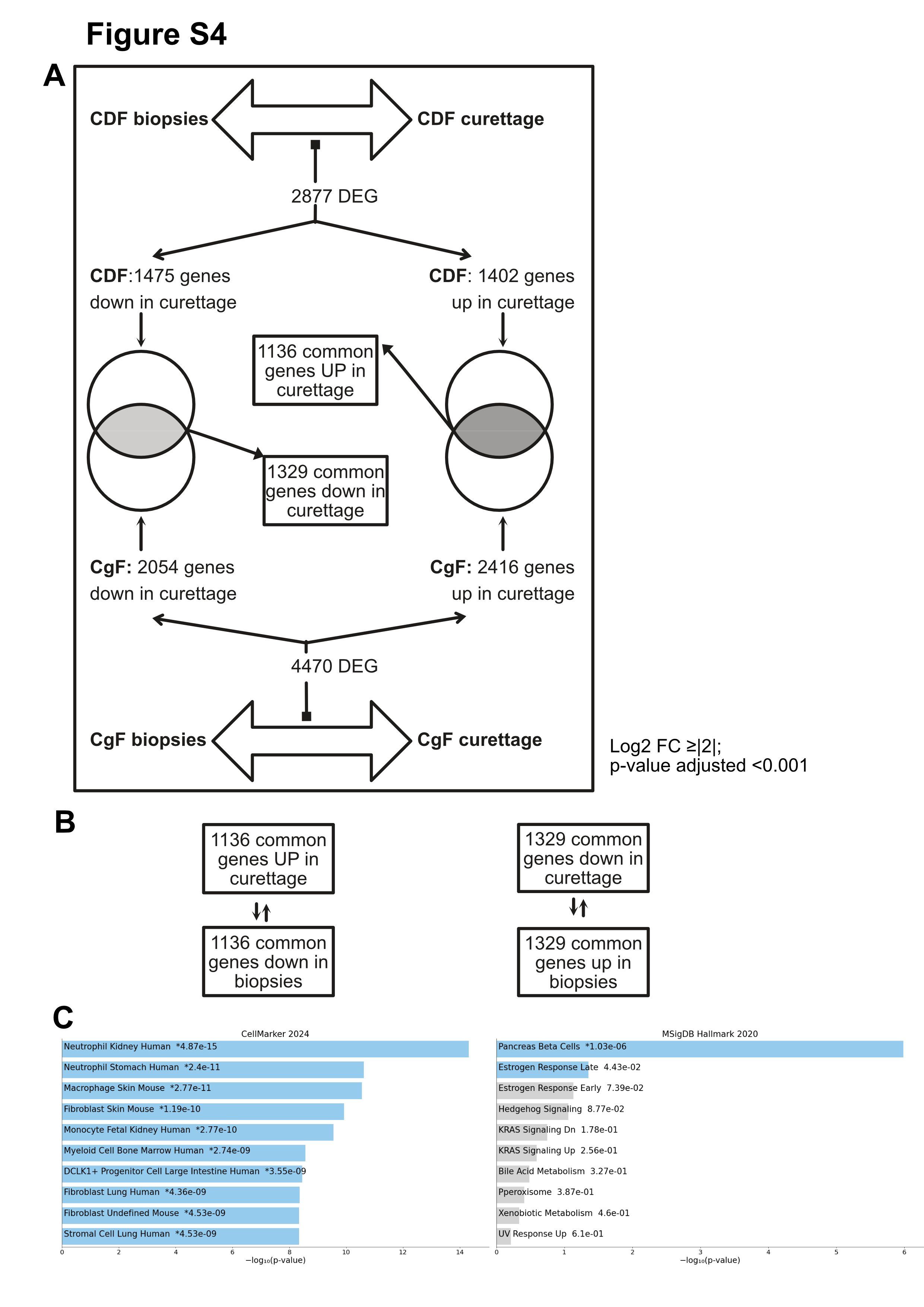

Supplement: jjag080_Supplementary_Data [file jjag080_supplementary_data.zip › Suppl_Fig_Tab_FISTULA_20260508_4.tiff]

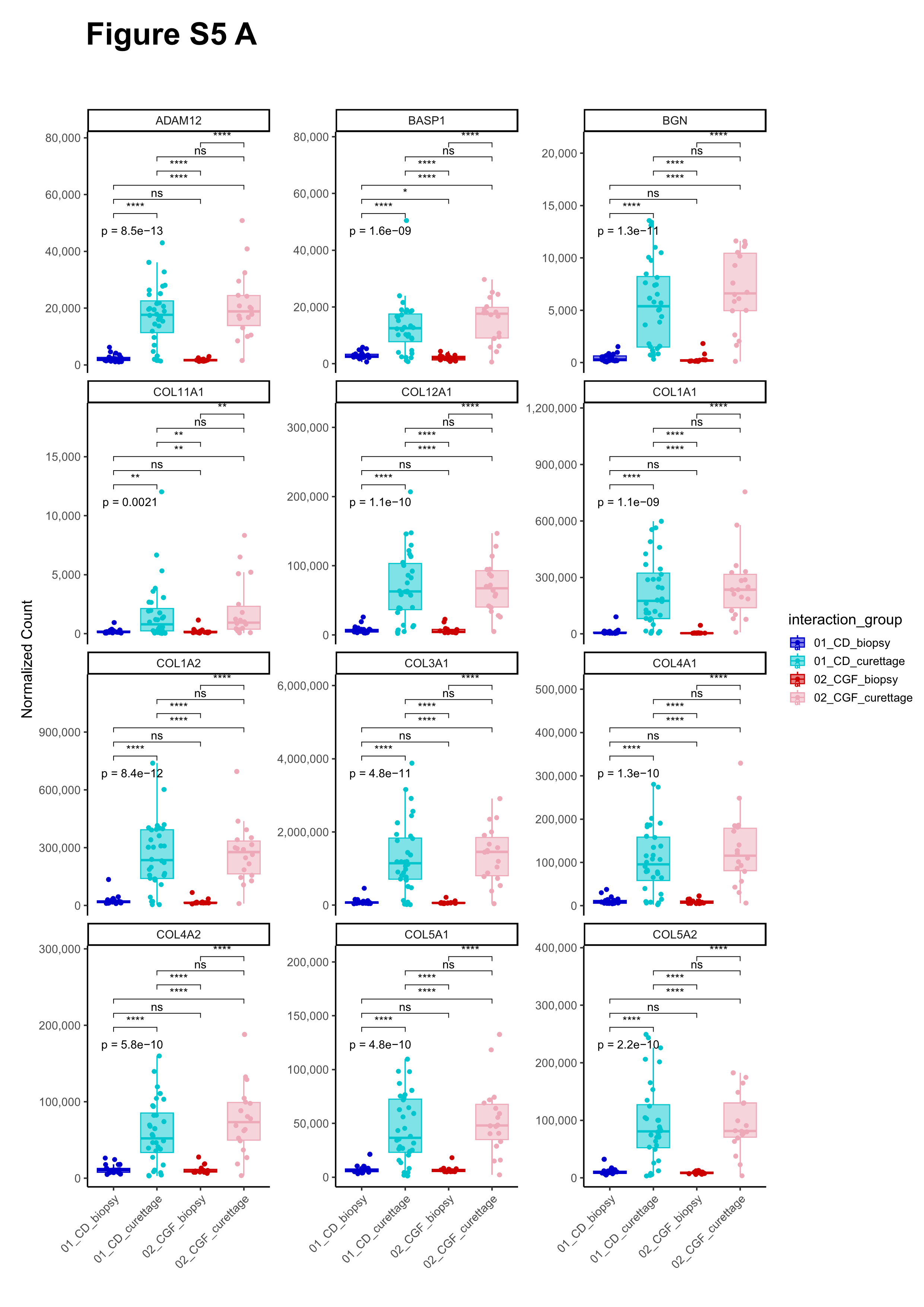

Supplement: jjag080_Supplementary_Data [file jjag080_supplementary_data.zip › Suppl_Fig_Tab_FISTULA_20260508_5.tiff]

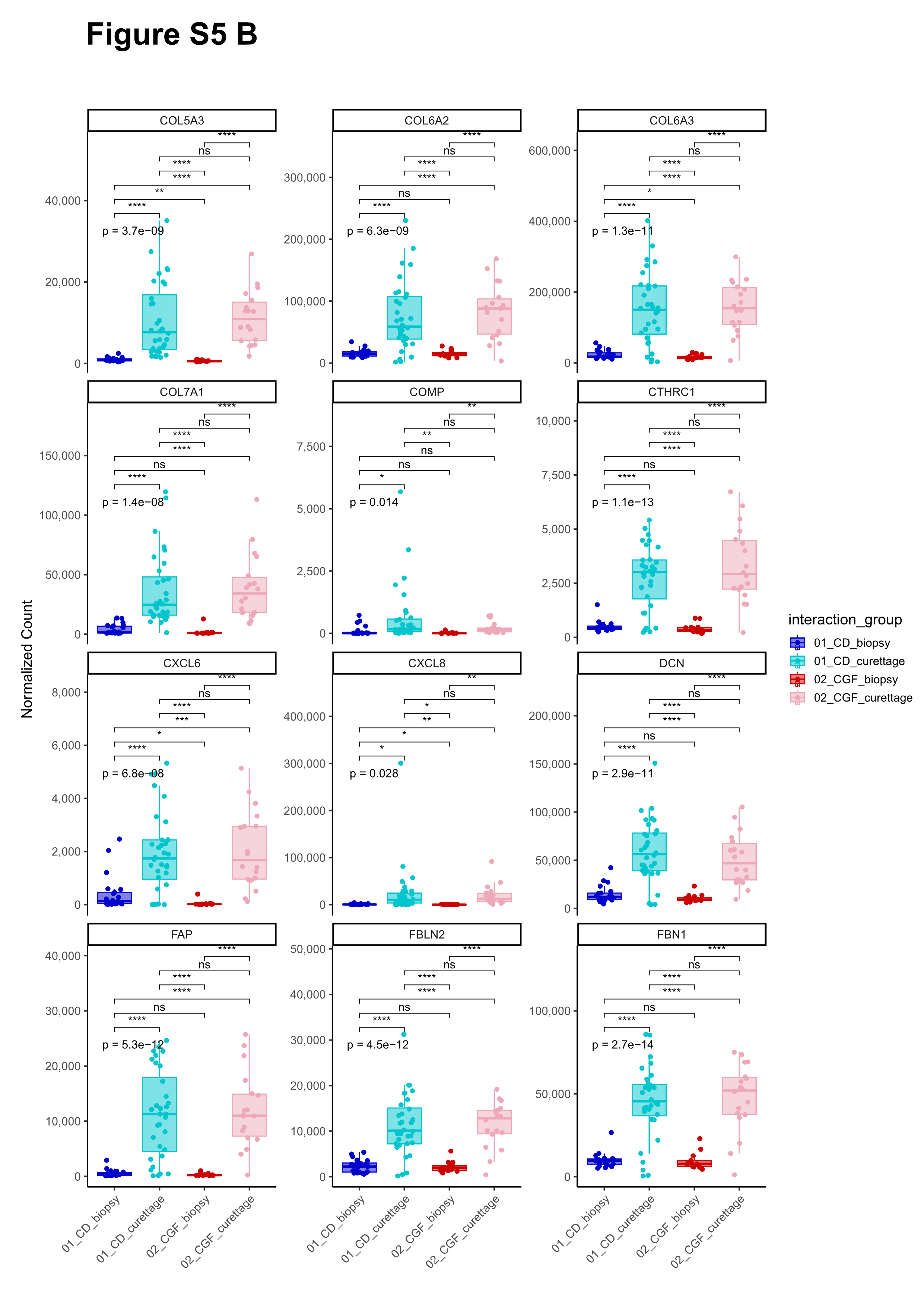

Supplement: jjag080_Supplementary_Data [file jjag080_supplementary_data.zip › Suppl_Fig_Tab_FISTULA_20260508_6.tiff]

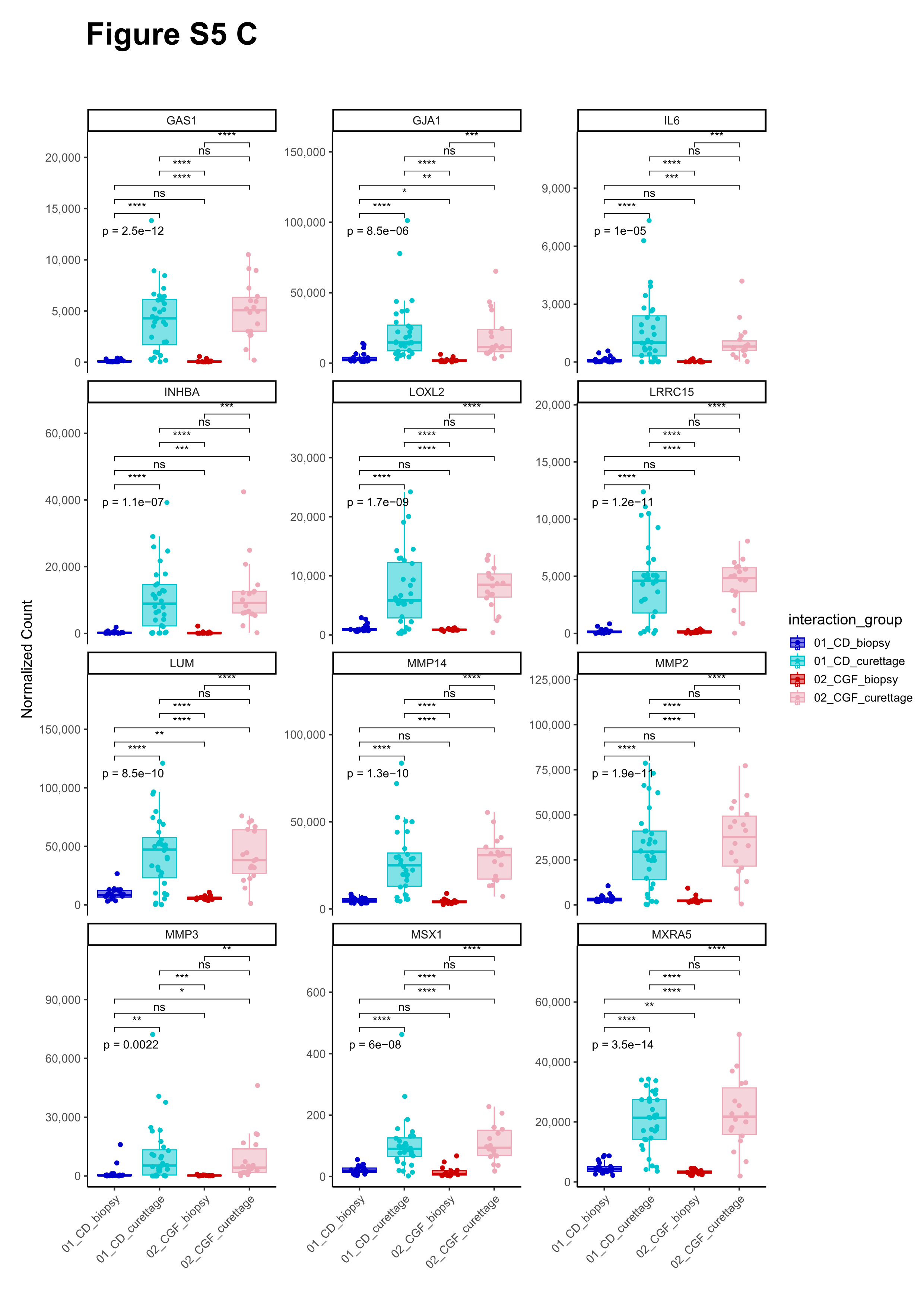

Supplement: jjag080_Supplementary_Data [file jjag080_supplementary_data.zip › Suppl_Fig_Tab_FISTULA_20260508_7.tiff]

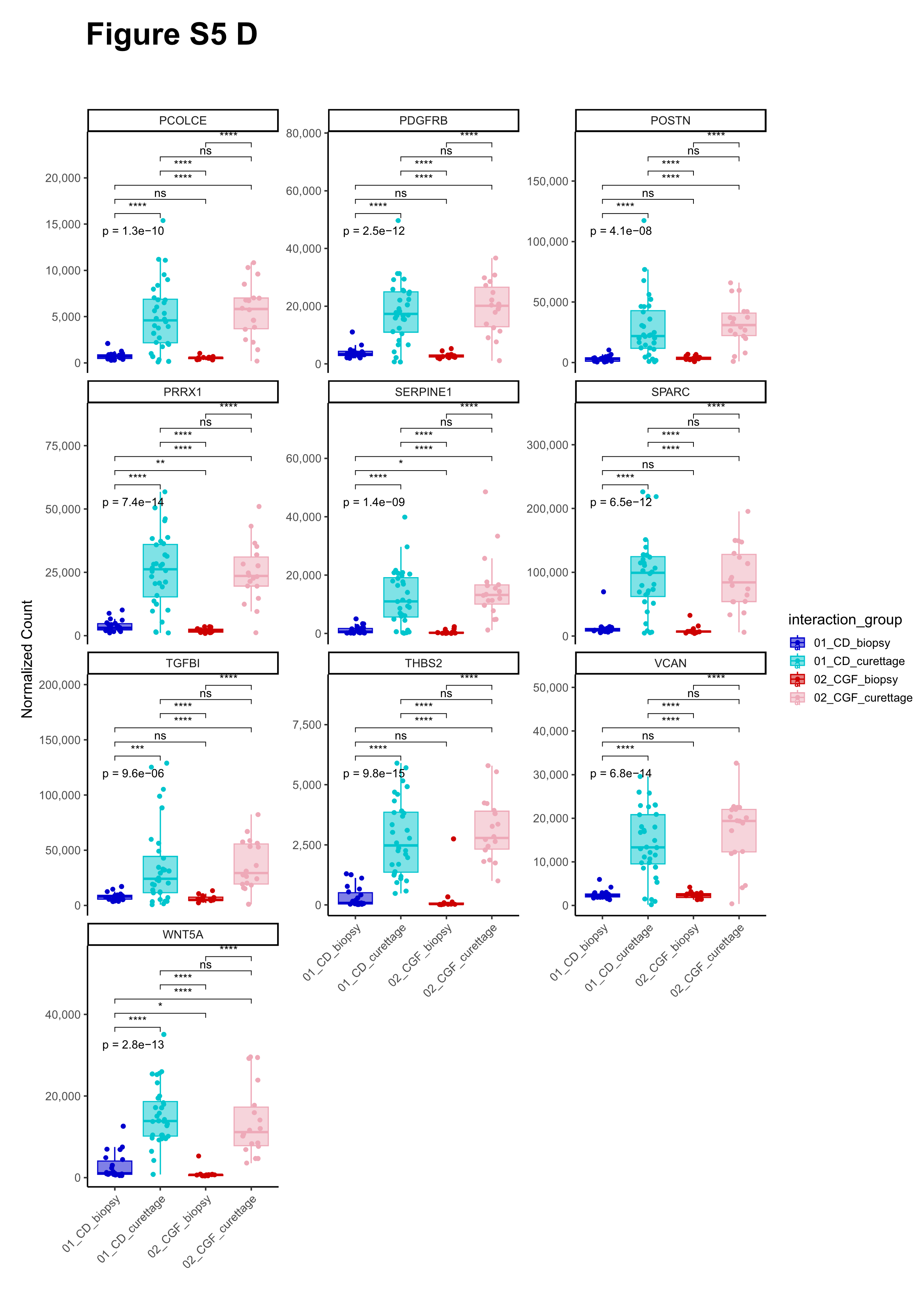

Supplement: jjag080_Supplementary_Data [file jjag080_supplementary_data.zip › Suppl_Fig_Tab_FISTULA_20260508_8.tiff]

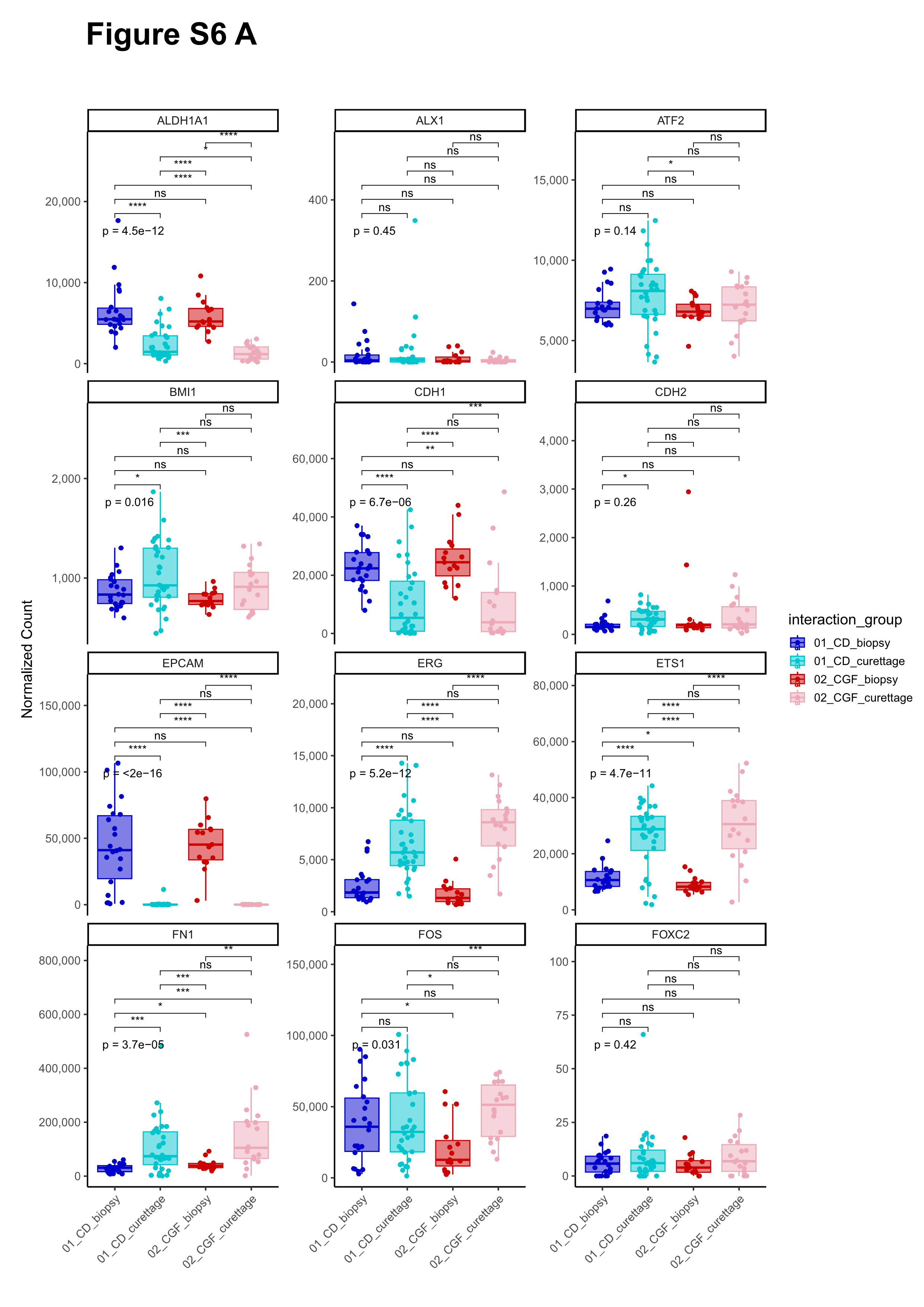

Supplement: jjag080_Supplementary_Data [file jjag080_supplementary_data.zip › Suppl_Fig_Tab_FISTULA_20260508_9.tiff]

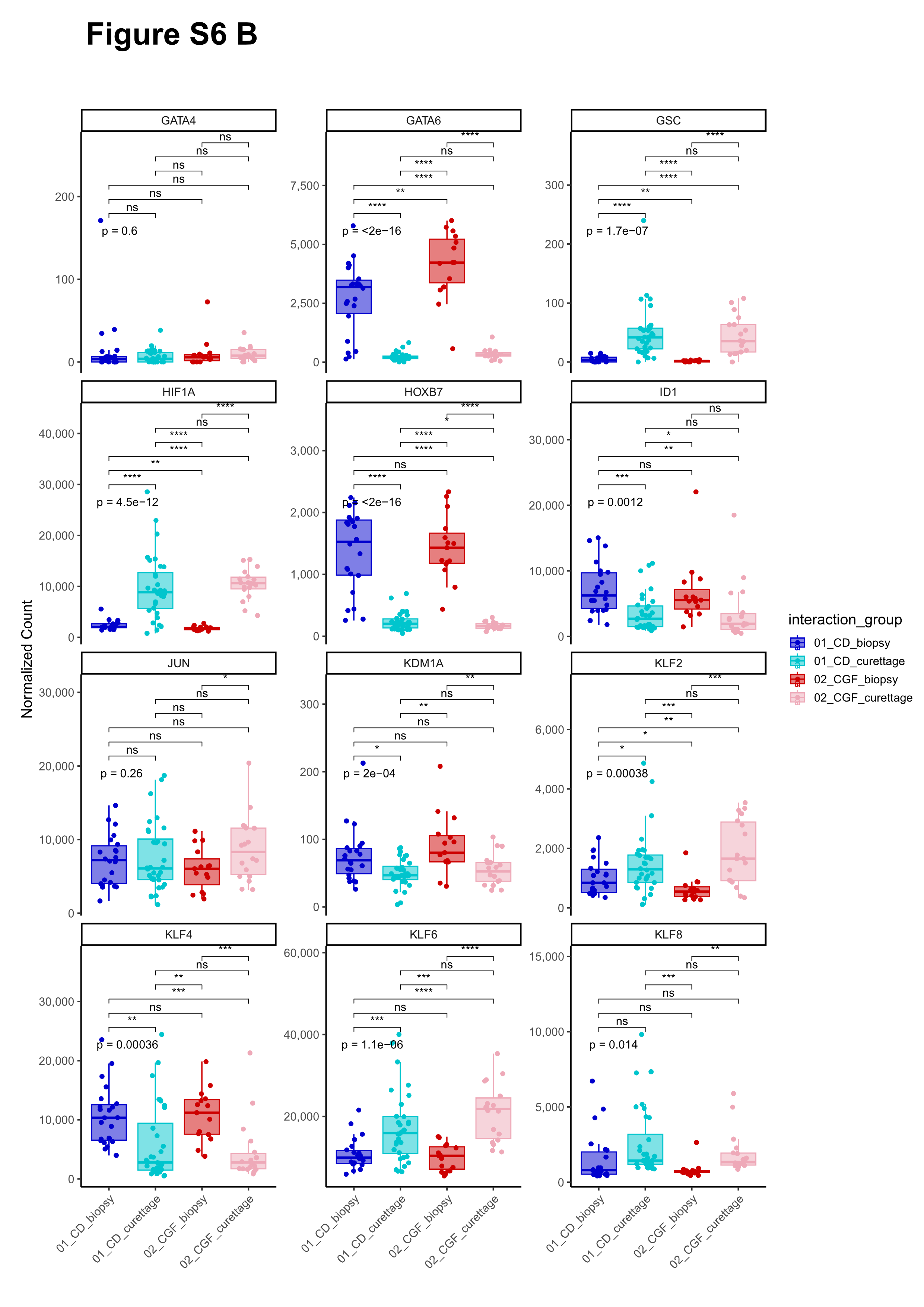

Supplement: jjag080_Supplementary_Data [file jjag080_supplementary_data.zip › Suppl_Fig_Tab_FISTULA_20260508_10.tiff]

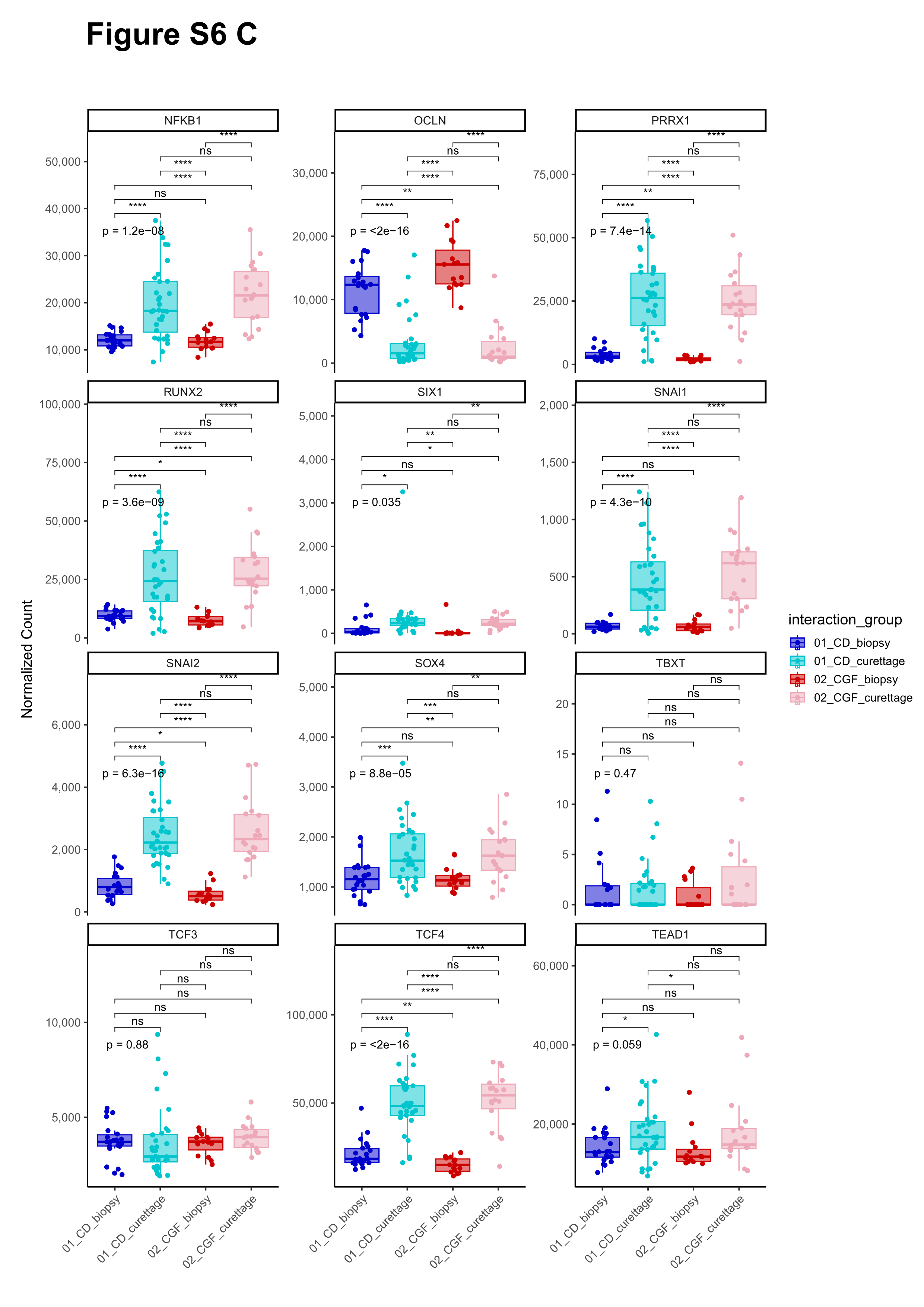

Supplement: jjag080_Supplementary_Data [file jjag080_supplementary_data.zip › Suppl_Fig_Tab_FISTULA_20260508_11.tiff]

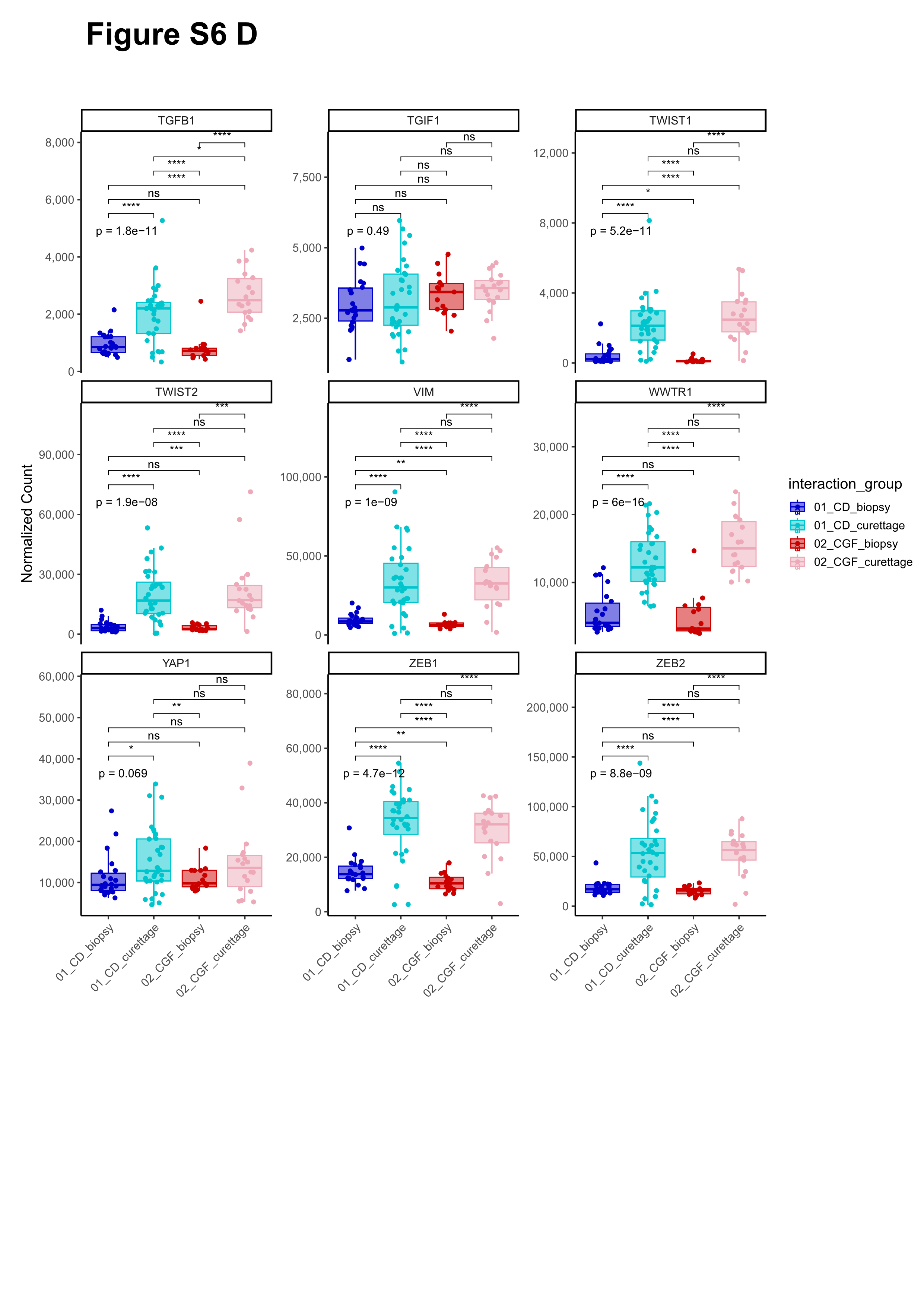

Supplement: jjag080_Supplementary_Data [file jjag080_supplementary_data.zip › Suppl_Fig_Tab_FISTULA_20260508_12.tiff]

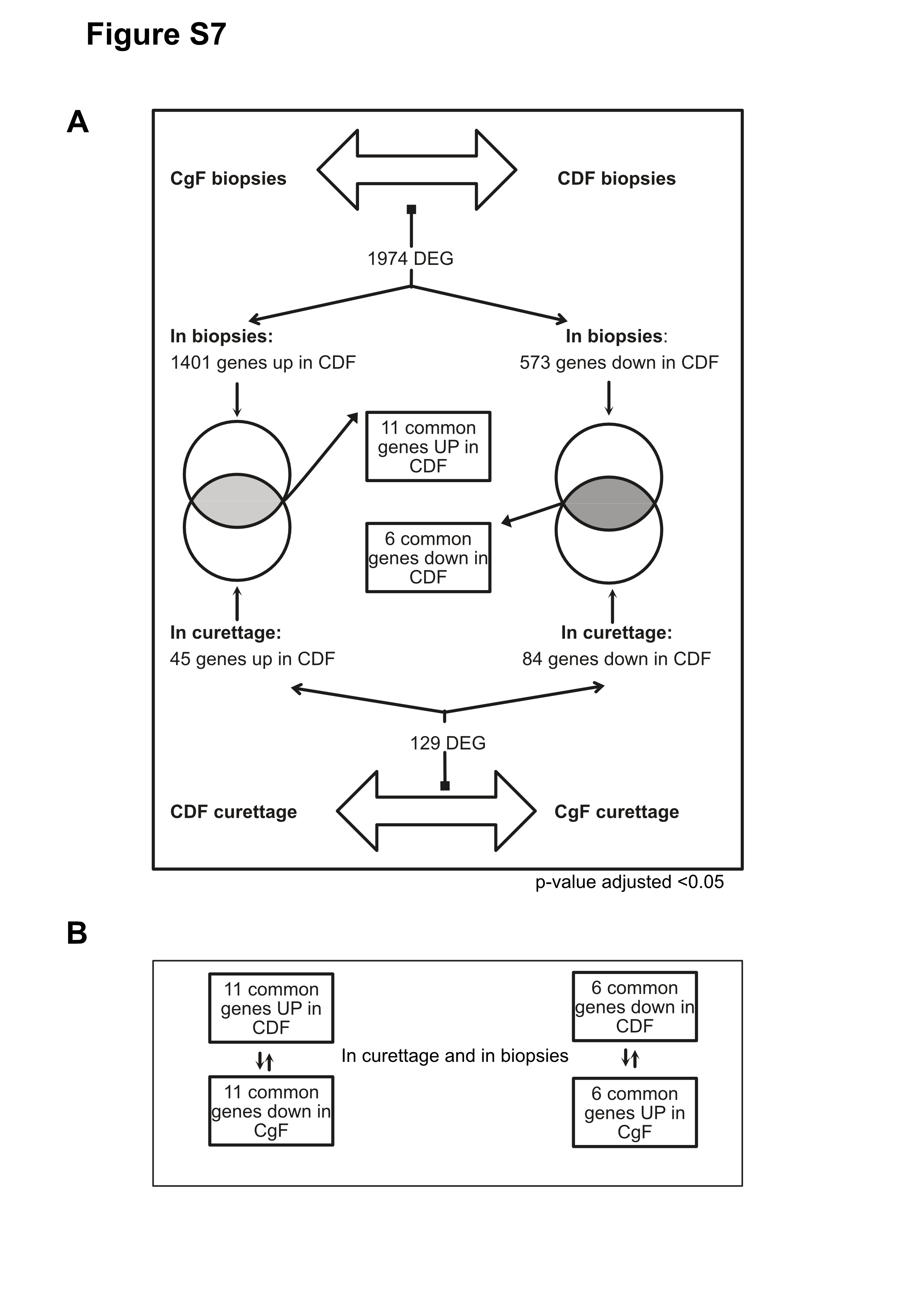

Supplement: jjag080_Supplementary_Data [file jjag080_supplementary_data.zip › Suppl_Fig_Tab_FISTULA_20260508_13.tiff]

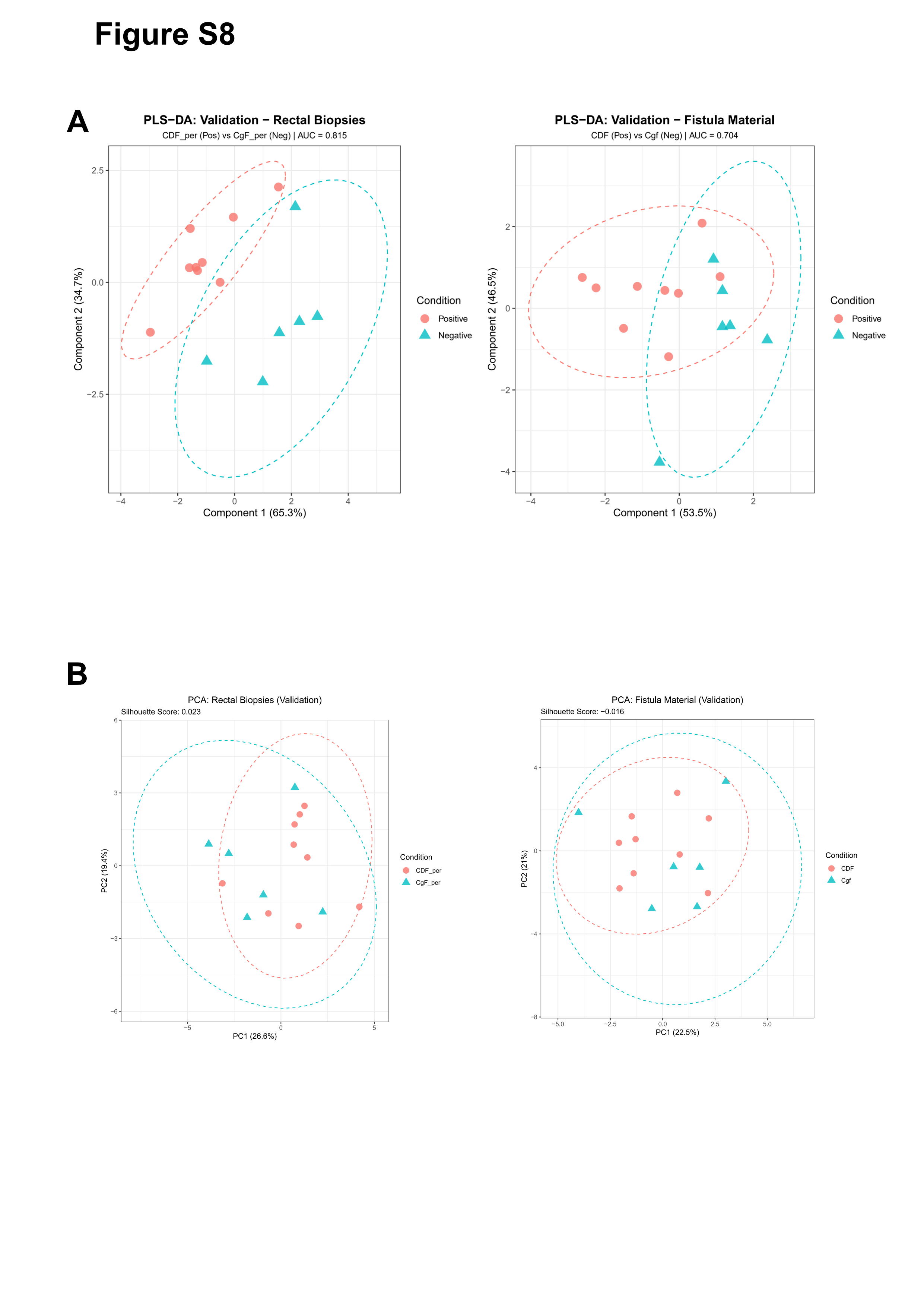

Supplement: jjag080_Supplementary_Data [file jjag080_supplementary_data.zip › Suppl_Fig_Tab_FISTULA_20260508_14.tiff]
